# An adapted model predictive control MPPT for validation of optimum GMPP tracking under partial shading conditions

**DOI:** 10.1038/s41598-024-59304-z

**Published:** 2024-04-24

**Authors:** Muhammad Abu Bakar Siddique, Dongya Zhao, Ateeq Ur Rehman, Khmaies Ouahada, Habib Hamam

**Affiliations:** 1 College of New Energy, China University of Petroleum (East China), Qingdao, 266580 China; 2 School of Computing, Gachon University, Seongnam, 13120 Republic of Korea; 3 Department of Electrical and Electronic Engineering Science, School of Electrical Engineering, University of Johannesburg, Johannesburg, 2006 South Africa; 4 Faculty of Engineering, Université de Moncton, Moncton, NB E1A3E9 Canada; 5 Hodmas University College, Taleh Area, Mogadishu, Somalia; 6 Bridges for Academic Excellence, Tunis, Centre-Ville Tunisia

**Keywords:** Maximum power point tracking (MPPT), Model predictive control, Optimal control, Renewable energy, Power converters, Energy conversion, Electrical and electronic engineering, Energy infrastructure, Renewable energy

## Abstract

The energy generation efficiency of photovoltaic (PV) systems is compromised by partial shading conditions (PSCs) of solar irradiance with many maximum power points (MPPs) while tracking output power. Addressing this challenge in the PV system, this article proposes an adapted hybrid control algorithm that tracks the global maximum power point (GMPP) by preventing it from settling at different local maximum power points (LMPPs). The proposed scheme involves the deployment of a 3 × 3 multi-string PV array with a single modified boost converter model and an adapted perturb and observe-based model predictive control (APO-MPC) algorithm. In contrast to traditional strategies, this technique effectively extracts and stabilizes the output power by predicting upcoming future states through the computation of reference current. The boost converter regulates voltage and current levels of the whole PV array, while the proposed algorithm dynamically adjusts the converter's operation to track the GMPP by minimizing the cost function of MPC. Additionally, it reduces hardware costs by eliminating the need for an output current sensor, all while ensuring effective tracking across a variety of climatic profiles. The research illustrates the efficient validation of the proposed method with accurate and stable convergence towards the GMPP with minimal sensors, consequently reducing overall hardware expenses. Simulation and hardware-based outcomes reveal that this approach outperforms classical techniques in terms of both cost-effectiveness and power extraction efficiency, even under PSCs of constant, rapidly changing, and linearly changing irradiances.

## Introduction

Recently, the escalating consumption of hydrocarbon fuels has led to significant environmental pollution, thereby making renewable energy sources a more appealing choice for electricity generation due to their sustainability and eco-friendliness. Solar energy stands out as a crucial renewable energy source and has found extensive application in photovoltaic (PV) power generation^1^. Nevertheless, the efficiency and cost challenges associated with PV systems have posed obstacles to the progress of PV power generation. Given that the performance of PV panels is influenced by external factors such as solar irradiance and temperature, the maximum power point (MPP) of a PV panel fluctuates in response to these external variables. Consequently, an effective maximum power point tracking (MPPT) technique becomes paramount in enhancing the efficiency of PV power generation systems.

**Figure 1:**
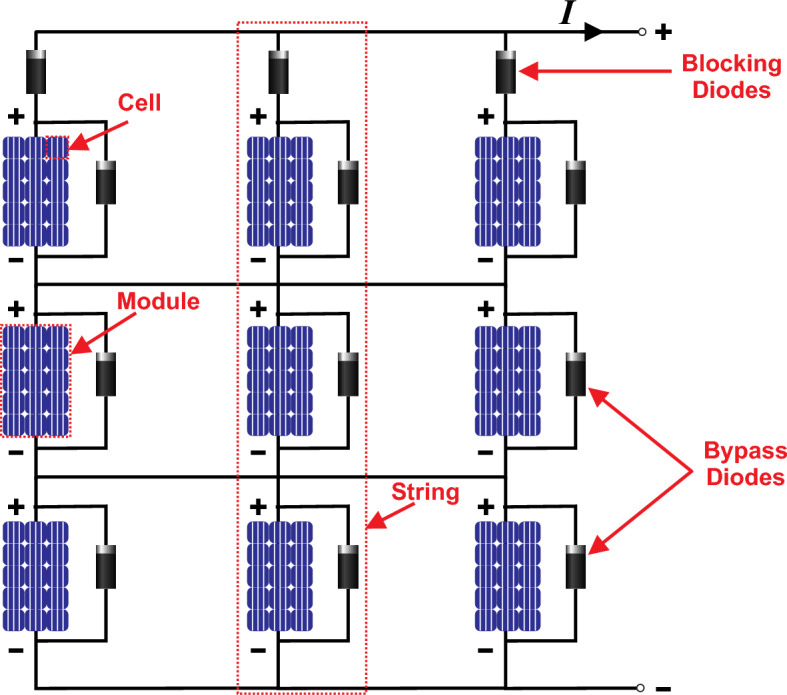
Architecture of 3 × 3 PV array.

**Figure 2:**
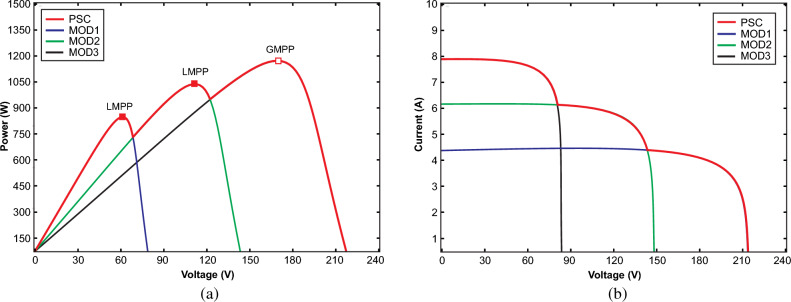
PV characteristics of 3 modules in a string under PSC (**a**) Power-voltage (**b**) current–voltage.

In a PV system, each PV array comprises numerous PV panels interconnected in both series and parallel configurations to achieve higher voltage and current levels, to maximize the output power of the PV system, as illustrated in Fig. 1. Within a solar power system, two types of diodes play distinct roles: bypass diodes and blocking diodes. Blocking diodes are employed to prevent the reverse flow of electricity. On the other hand, bypass diodes are utilized to mitigate the adverse effects of shading and prevent localized overheating, ultimately reducing power losses. This scenario is referred to as partial shading conditions (PSC), wherein each panel may be subjected to varying levels of solar irradiation and temperature simultaneously^2^. Unlike traditional MPPT techniques, which consider the optimization of standalone individual PV modules or strings, global maximum power point tracking (GMPPT) considers the effects of shaded and unshaded modules within a PV array. Table 1 explains the comparative analysis and major challenges of GMPPT over MPPT schemes. When a PV array operates under PSCs, its power voltage (P–V) characteristic curve exhibits multiple peaks. Among these peaks, only one is recognized as the Global Maximum Power Point (GMPP), while the others are termed Local Maximum Power Points (LMPPs) as presented in Fig. 2. GMPPT aims to ensure the overall maximum output power by finding the GMPP of the entire array and prevents the tracking of LMPPs. In the existing literature, a multitude of GMPPT algorithms have been introduced to effectively trace and control the GMPP of PV systems under PSCs^3^.

**Table 1:** Comparative analysis and major challenges of GMPPT over MPPT algorithms.

MPPT	GMPPT	Major challenges
Optimizes the power generation of individual PV module or string	Maximizes the total generated output power of the entire PV array	GMPPT algorithms often demand more sensors and monitoring devices for collecting data from each PV module across the array
Focuses on adjusting the operating parameters i.e. voltage and current to follow the MPP along its current–voltage (I–V) curve	Determines the overall GMPP of the entire array and avoiding LMPPs through dynamic adjustment of the operating parameters of individual modules or strings	It requires more computational resources because they need to process data from multiple PV modules simultaneously
Operates under varying environmental factors such as temperature, irradiance and shading	Functions under complex environmental conditions of partial shading and non-uniform irradiance	Implementation of these algorithms comparatively can be challenging due to harsh and unpredictable climatic profiles
Algorithms have typically simple and traditional schemes	Approaches are comparatively advanced and have predictive models	It can face scalability issues as the size of the PV array increases

The impacts of shadow intensity, shadow velocity, and shadow size on PV systems have been investigated^4^. Furthermore, investigations have been conducted to understand the correlation between the maximum ramp rate of irradiance and power^5^. Current MPPT technologies can be classified into three distinct categories: traditional techniques, intelligent algorithms, and optimization-based control approaches.

## Related work

It is widely recognized that conventional MPPT algorithms, including the perturb and observe (P&O) algorithm, incremental conductance (IC)^6^, and constant voltage method (CVM), face limitations in effectively following the GMPP under PSC^7^. In response to this challenge, numerous alternative algorithms have been put forth to address the GMPP tracking issue^8^. Among these approaches^9^, intelligent control methods utilizing fuzzy logic control (FLC)^10,11^ and artificial neural networks (ANN)^12^ demand substantial input data. Optimization-based control strategies including particle swarm optimization (PSO)^13^, genetic algorithm (GA)^14^, firefly algorithm (FA)^15^, seagull optimization algorithm (SOA)^16^, artificial bee colony (ABC) algorithm^17^, and gray wolf optimization (GWO) algorithm^18^, model predictive control (MPC)^19^ and sliding mode control (SMC)^20^ approach have been harnessed for GMPP tracking. Due to their simplicity in design and implementation, MPC and its variations have been extensively explored for GMPP tracking in PV systems. A comprehensive review of different MPPT algorithms has been conducted under PSCs to track GMPP as presented in Table 2.

**Table 2:** Comprehensive review of GMPPT algorithms under PSCs.

Ref	Algorithm	Analysis
^27^	Modified P&O	The modified P&O algorithms is the simplest approach which do not require high computational resources, however, it suffers from oscillations around the GMPP as it perturbs the operating point without considering the system's voltage limits, especially under PSCs due to passing clouds
^7^	FOCV	The fraction used for voltage adjustment might not provide optimal accuracy in tracking the true GMPP under varying conditions because it only uses single loop consisting of feedback
^28^	FSCC	Selecting an appropriate fraction of the short circuit current using an additional switch is crucial, and it might not provide the precision and adaptability needed to consistently optimize GMPP in dynamic environments
^29^	IC	The performance of IC-GMPPT can depend on the accurate tuning of its parameters, such as the step size used in the calculations. Poor parameter tuning can lead to suboptimal operation
^10^	FLC	It can be challenging to set up proper membership functions and rules for optimal performance. It also requires significant computational resources to process its functions, potentially leading to higher hardware and implementation costs
^12^	ANN	Designing and training an effective ANN for GMPPT requires expertise in neural network architecture, training algorithms, and data preprocessing. This complexity can result in longer development times and higher implementation costs
^15^	FFA	It exhibits fast convergence towards GMPP, automatically preventing entrapment at LMPPs, and effectively reducing oscillations during tracking under PSCs. However, as the iterations progress, it may exhibit a tendency to cluster, resulting the complexity in computation
^14^	GA	GA involves several parameters that need to be tuned, such as population size, mutation rate, and crossover rate. Poor parameter choices can lead to slow convergence or premature convergence to suboptimal solutions
^13^	PSO	Implementation of PSO requires expertise in algorithm design, parameter tuning, and fitness function definition
^2^	OD-PSO	It presents rapid response by searching the small patch near to the GMPP under harsh environmental conditions. However, the measured output of each PV module should be used by the algorithm to dynamically adjust the operating point but this model is not considering the proper details of PV array which may compromise the overall tracking efficiency
^30^	MPC	MPC involves solving optimization problems using fixed and variable switching techniques. In fixed switching technique, a digital observer is used to forecast the operating points either voltage or current and variable switching method employs discrete-time model of the utilized converter to generate switching signal
^31^	IC-MPC	MPC is utilized by computation of reference current tracking using IC. A PI controller minimizes the error between reference current and actual inductor current. Similarly, two-stage algorithm segments current characteristics and maintains GMPP by minimizing the cost function. However, steady state response is poor while irradiance step changes
^32^	MMPC-SEPIC	It is capable of operating with both fixed and adaptive step sizes and effectively tracking under various climatic conditions. To enhance efficiency and minimize voltage stresses, a modified SEPIC is employed with reduced number of sensors in the power stage
Proposed	APO-MPC	Prevents the PV array from deviating into LMPPs during PSCs and enhance overall power generation efficiency. It achieves the GMPP by optimizing cost function using reference current of the PV system. This strategy can function with both constant and VSSs, offering effective tracking of the GMPP across a range of climatic profiles

A centralized MPPT controller based on PSO was proposed for multi-module PV systems equipped with multiple converters^21^.The overall distribution (OD) approach is introduced to obtain the nearest position to the GMPP rapidly under PSCs and combined with PSO to enhance the accuracy^2^. The direct duty cycle control (DDCC) method employed the hybrid P&O-based fractional open circuit voltage (FOCV) algorithm to regulate the duty cycle of the pulse-width modulation (PWM) signal, thereby eliminating the need for proportional-integral control loops^22^. The traditional PSO was altered by linearly reducing the inertia weight and the cognitive parameter while linearly increasing the social parameter^23^. This adjustment demonstrated the capacity to achieve the GMPP with fewer iterations. Conversely, the swarm size was systematically reduced as it approached the GMPP^24^. An innovative adaptive strategy for PSO is introduced to pursue the GMPP in PV system^25^. This approach effectively addresses issues related to PSO for GMPP, including the initialization of particle values.

GMPPT techniques based on PV array models have the primary objective of tracking reference values through dynamic adjustment of the operating points of individual PV modules. To achieve this, a model for the GMPP reference value has been introduced to estimate it without the time-consuming process of iterative control^26^.

The concept of a Fractional Characteristic Curve (FCC) derived from a two-diode model has been proposed for estimating GMPP reference values^33^. Mathematical models specifically designed for partial shading scenarios have been studied to calculate voltage reference values, both LMPP and GMPP^34^. Utilizing a parabolic curve to approximate the partial I–V characteristic curve between the MPP and the open-circuit voltage point has been applied to determine the GMPP reference value^35^. Voltage ripple in PV systems has been addressed by regulating the input capacitor^36^. Additionally, an approximation function, constructed based on six sampling points, has been developed to match the I–V characteristic curve and estimate the GMPP under various irradiance conditions^37^. A customized hybrid GMPPT approach that combines an ANN with a modified P&O technique is introduced^38^. It indirectly derives the illumination intensity for each module within the PV array by capturing specific data points using cost-effective voltage-current sensors.

An MPC approach, designed with optimized dynamic process characteristics, has been applied to enhance the optimization of the PV MPPT process^39^. Due to the robust computing capabilities of the field programmable gate array (FPGA), the MPC asserts swift dynamic responses and a notable degree of stability. Furthermore, the MPC demonstrates proficiency in swiftly adapting to minimal oscillations in both dynamic and steady-state situations, ensuring a prompt dynamic response and commendable stability, even in the presence of rapidly changing weather conditions^40^. The MPC stands out due to various crucial characteristics, including discrete switch implementation, the management of multiple variables, the incorporation of nonlinear constraints on controlled variables within a unified objective function, and the ability to swiftly track reference values for each PV module within the array^41^. By using the average current control method, a reference current value based on the desired output power or GMPP is computed based on the system’s operating conditions i.e. irradiance and temperature, and controller aligns the actual current supplied by PV system with this reference current. MPC controller utilizes this reference current to predict the control inputs for converter to optimize the actual current value and maintains the GMPP under diverse weather profiles^42^. It continuously revises its forecasts and control actions by incorporating real-time data from sensors in the form of feedback to verify whether the system is operating at the GMPP. If it deviates or settles in LMPP, the reference current is adjusted accordingly to re-align the system on track. Thus, the use of feedback mechanism and continuous adjustments of the reference current value under PSCs, the algorithm ensures the GMPP tracking even in the presence of LMPPs^31^. The proposed hybrid algorithm in this study has been devised as an enhancement to the traditional P&O and classical MPC method. This decision was driven by the fact that P&O is frequently employed as a foundational approach for new algorithms, owing to its straightforward implementation. Similarly, the MPC approach is renowned for precise and accurate tracking capabilities. Therefore, this research study has mainly focused on enhancing the P&O and MPC algorithm's performance by incorporating adapted adjustments and utilizing their hybrid combination.

In this study, a hybrid APO-MPC approach is proposed to prevent the settlement of PV system into LMPPs during PSCs and enhance overall generation efficiency. The proposed algorithm achieves the GMPP by optimizing the cost function using the reference current of the PV system which also reduces the requirement of the output-side current sensor. The APO strategy functions with variable step sizes (VSSs) and offers reference current trajectory for rapid tracking of the GMPP across a range of climatic profiles. The significant contributions of this study are outlined as follows.

i. CMPC-based MPPT algorithm solely relies on the converter's parametric model which can only perform single-step predictions. On the other hand, the proposed APO-MPC incorporates the modified model of the boost converter and dynamically updates the system parameters to enable upcoming next-step predictions for PV systems.
ii. Existing MPPT algorithms based on deep neural networks (DNNs) require retraining of the system model under varying weather conditions. However, this study optimizes GMPP under PSCs using a cost function minimization scheme.
iii. Traditional MPC-based MPPT techniques typically employ a single-stage optimization approach. However, the proposed strategy introduces a two-stage optimization process. The first stage targets the computation of reference current, while the second stage focuses on the optimization of optimum output power. This two-stage optimization process enhances the tracking speed of the system.
iv. Earlier versions of MPC were limited to achieving MPP under uniform irradiance conditions (UICs). However, this work was implemented on various climatic profiles under PSCs.

**Figure 3:**
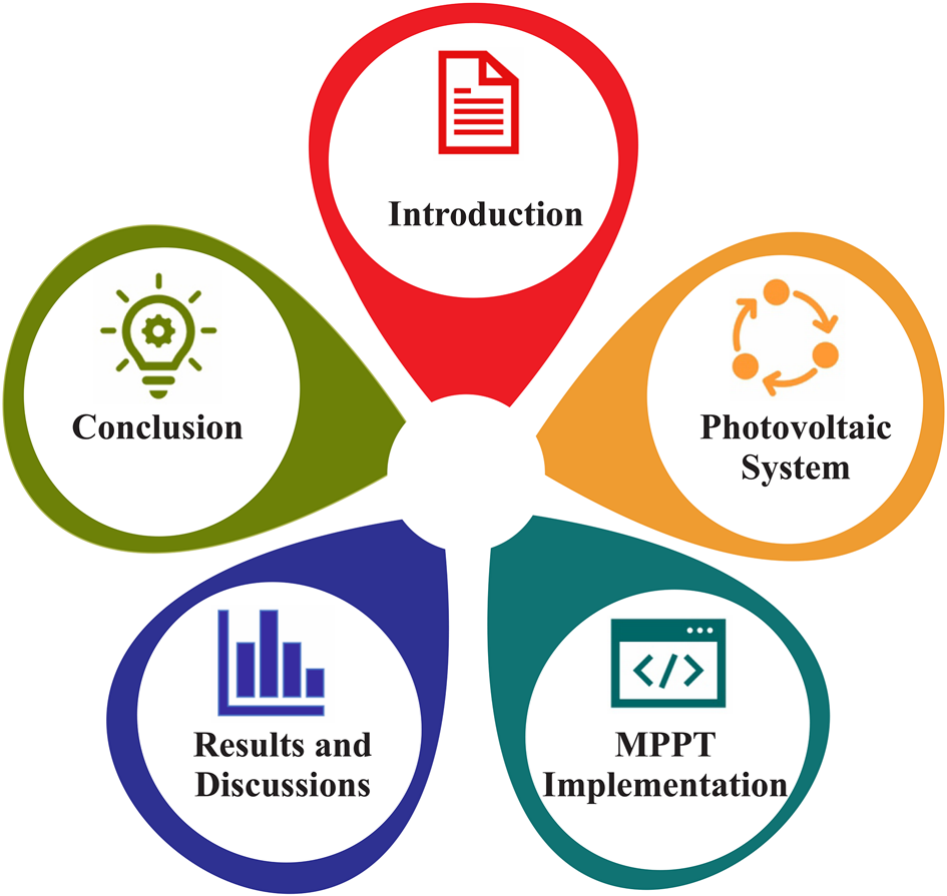
Section-wise research distribution.

Figure 3 illustrates the structure of this study. Photovoltaic System elaborates on the PV array design and characteristics under PSCs. Further, it also incorporates modeling of the modified boost converter. In MPPT Implementation, various MPPT algorithms are briefly discussed and an adapted hybrid algorithm is proposed. Results and Discussions evaluates and validates the implemented algorithm in MATLAB Simulink and experimental setup under various weather scenarios of PSCs. Finally, the paper concludes in Conclusion.

## Photovoltaic system

In this section, we delve into the modeling of the components of the two-stage PV system, which includes the PV array and the modified boost converter.

### Modeling of PV array

The PV array is characterized using the single-diode model, providing an accurate representation of the output characteristics for a variety of PV cells and modules ^9,43–46^. This model consists of essential components, including a photocurrent replaced with current source ${I}_{ph}$, a diode *D*, a parallel resistance ${R}_{p}$, and a series resistance ${R}_{s}$. The total module output current ${I}_{pv}$ can be expressed as follows:

1 $$I_{{pv}} = I_{{ph}} - I_{o} \left[ {exp\left( {\frac{{V + IR_{s} }}{{A_{i} V_{{th}} }}} \right) - 1} \right] - \frac{{V_{{pv}} + I_{{pv}} R_{s} }}{{R_{p} }}$$

where ${I}_{o}$ is the diode leakage current, ${V}_{th}$ is the thermal voltage at actual temperature ${T}_{a}$ which is given by ${V}_{th}={{K}_{B}{T}_{a}}/{q}$, ${A}_{i}$ is the ideality factor of diode D and *q* is the electronic charge and ${K}_{B}$ is Boltzmann constant.

**Figure 4:**
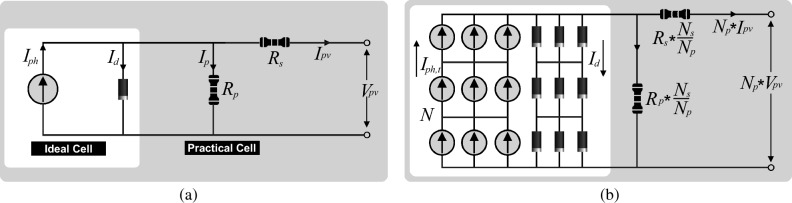
Ideal and practical single diode model (**a**) PV cell (**b**) PV array.

The PV cells are configured in different series and parallel combinations to maximize the output power and configure a PV module. Various PV strings consist of series modules make a PV array. Figure 4 provides a single diode model of a PV cell and an array.

2 $${I}_{pv}={I}_{ph}{N}_{p}-{I}_{o}{N}_{p}\left[exp\left(\frac{V+I{R}_{s}}{{{N}_{s}{A}_{i}V}_{th}}\right)-1\right]-\frac{{V}_{pv}+{I}_{pv}{R}_{s}}{{R}_{p}}$$

where ${N}_{p}$ is the number of cells connected in parallels and ${N}_{s}$ is the number of series-connected PV cells. Although, *R*_*s*_ has an extremely small value and *R*_*p*_ has an exceptionally large value. After simplification, both of the resistors would be neglected.

In above mentioned PV array model, the photocurrent of the PV cell may be computed approximately by defining its dependency on solar irradiance and temperature using Eq. (3).

3 $${I}_{ph}=\frac{{G}_{a}}{{G}_{r}}\left[{I}_{sc}+{K}_{sc}({T}_{a}-{T}_{r})\right]$$

where ${G}_{a}$ is actual solar irradiance, ${G}_{r}$ is reference solar irradiance at standard testing conditions (STC), ${K}_{sc}$ is temperature coefficient of PV cell under short circuit and ${T}_{r}$ is reference temperature at STC.

4 $${I}_{o}={I}_{rs}{\left(\frac{{T}_{a}}{{T}_{r}}\right)}^{3}exp\left[\left(\frac{{qE}_{g}}{{A}_{i}{K}_{B}}\right)\left(\frac{1}{{T}_{r}}-\frac{1}{{T}_{a}}\right)\right]$$

where ${E}_{g}$ is the energy band gap and ${I}_{rs}$ is the diode reverse saturation current given by Eq. (5).

5 $${I}_{rs}=\frac{{I}_{sc}}{exp\left[\left(\frac{{V}_{oc}}{{N}_{s}{V}_{th}}\right)-1\right]}$$

When the PV array operates under UIC, it exhibits a single MPP in its resulting P–V characteristic curve. However, under PSCs, multiple LMPPs appear in the P–V curves. This can be mainly attributed to the inclusion of bypass diodes linked in parallel with each PV module. When PV modules are connected in parallel, shaded strings draw current from other parallel strings, resulting in a circulating current that diminishes the efficiency of the PV panel. To counter this, blocking diodes, as depicted in Fig. 1, are employed in each series string. These blocking diodes ensure that current flows solely out of the series array into the external circuit. It's noteworthy that the GMPP may occur within either the lower or higher voltage range, depending on specific irradiation conditions. This variability poses a challenge for the direct application of conventional MPPT algorithms.

### PV characteristics under PSC

In any PV array, two fundamental factors play a crucial role in any analysis: current and voltage. Solar power output is the result of the interaction between these two factors. There's a direct relationship between the sunlight projected onto PV cells and the generation of electric current. It's important to note that, in no-load conditions, all of the current flows through the diode *D*. The series and shunt resistors are responsible for heat-dissipating voltage drops and leakage losses, respectively. As the output load increases, the output current also increases proportionally. Consequently, the output current *I* reaches its maximum peak, while the open-circuit voltage experiences a minimum. With changing climate conditions, the power point shifts from the MPP to the right and left. This study focuses on the discussion of how the MPP shifts based on these climate-induced changes.

**Figure 5:**
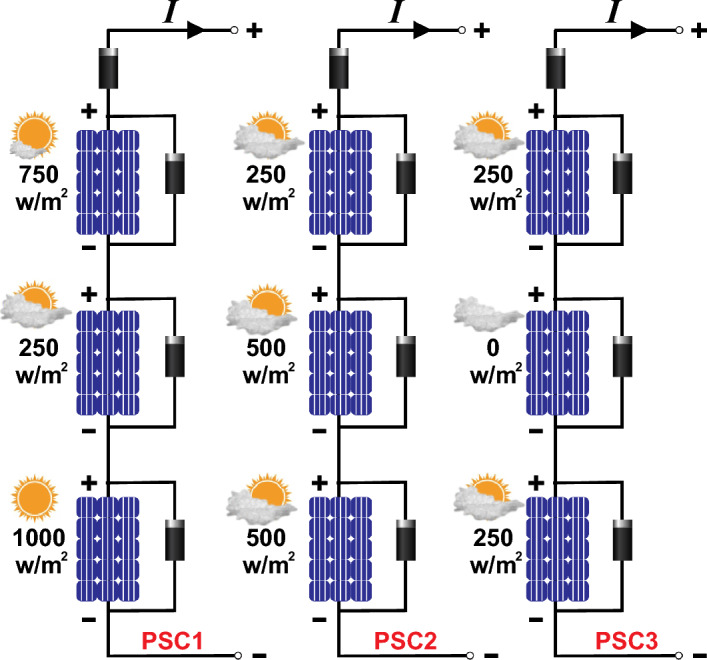
PV strings under PSC with different irradiances.

**Figure 6:**
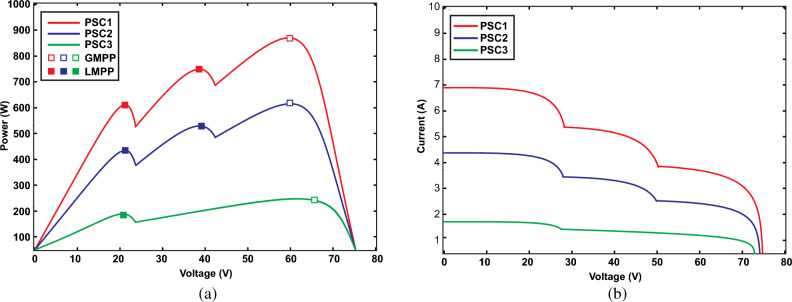
Characteristics of PV array under PSC with different irradiances (**a**) Power-voltage (**b**) Voltage-current.

In case of PSC, the PV module experiences varying irradiance levels, causing certain cells within the module to receive different levels of sunlight compared to others. These shaded segments within the PV string generate lower current, yet it's crucial for current to remain consistent in a series-connected PV system. However, under such circumstances, the shaded module operates in a reverse-biased state, resulting in a significant voltage drop across the shunt resistance *R*_*sh*_. To mitigate the substantial power loss associated with PSC, a bypass diode is employed. The role of the bypass diode is to minimize the adverse effects of the shaded module by introducing a mere 0.7V drop. Suppose, PV array consists of N_ser_ × N_par_ modules denoted as PV_xy_ where x is number of parallel strings and y is the number of series modules in a string. The computation of overall current and voltage of array is given by Eq. (6) ^32^. Figure 5 illustrates the behavior of the PV strings under PSC where different irradiance levels affect various PV modules. While PSC, shaded modules act as open circuits, and all current flows through the shunt resistor *R*_*sh*_, leading to a significant voltage drop. The bypass diode functions in a forward-biased state to counteract this substantial voltage drop. The P–V characteristics curve represents PSC scenarios with multiple peaks as depicted in Fig. 6.

6 $$\left\{\begin{array}{c}{I}_{arr}=\sum_{x=1}^{{N}_{par}}{I}_{xy}\\ {V}_{arr}=\sum_{y=1}^{{N}_{ser}}{V}_{xy}\\ {G}_{arr}=\sum_{x=1}^{{N}_{par}}\left(\sum_{y=1}^{{N}_{ser}}{G}_{xy}^{-1}\right)\end{array}\right.$$

where I_arr_ is the overall current of PV_xy_ with parallel connected N_par_ modules, V_arr_ is the overall voltage across PV_xy_ with series connected N_ser_ modules and G_arr_ is the overall conductance of PV_xy_.

Each curve includes a primary peak referred to as the GMPP, and any additional peaks on the same curve are termed LMPPs. For instance, in the curve of PSC1, the GMPP is 815W, with LMPP1 at 490W and LMPP2 at 405W. Similarly, the second curve of PSC2 features a GMPP of 500W, with LMPP1 at 380W and LMPP2 at 197W. The third curve of PSC3 has a GMPP of 182W, with LMPP1 at 95W. To effectively locate the GMPP from the different LPs under PSC, an efficient algorithm is essential^47^. The parameters of the PV array are computed as illustrated in Table 3 under various climatic profiles as demonstrated in Table 4.

**Table 3:** Parameters and characteristics of PV array under PSC.

Parameter	Symbol	Value
Short-circuit current of PV panel	*I*_*sc*_	23.52 A
Open circuit voltage of PV panel	*V*_*oc*_	217.5 V
PV current at maximum power point	*I*_*mpp*_	19.83 A
PV voltage at maximum power point	*V*_*mpp*_	186 V
Parallel strings in the PV array	*No*	3
Series-connected modules per string	*No*	3
Cells per PV module	*N*_*cell*_	60
Maximum Power of PV panel	*P*_*mp*_	3688.2 W
Temperature coefficient of V_oc_	*K*_*oc*_	− 0.36%/deg C
Temperature coefficient of I_sc_	*K*_*sc*_	0.10%/deg C
Diode ideality factor	${A}_{i}$	1.968
Parallel resistance	*R*_*p*_	65.1984 Ω
Series resistance	*R*_*s*_	0.1307 Ω
Diode saturation current	*I*_*s*_	2.819e^−10^
Energy band gap	${E}_{g}$	1.12–1.15 eV
Boltzmann constant	${K}_{B}$	1.38 × 10^–23^ J/K
Solar irradiance at STC	${G}_{r}$	1000 W/m^2^
Temperature at STC	${T}_{r}$	25 ℃
Electronic charge	Q	1.6 × 10^–19^ C

**Table 4:** PV strings with different solar irradiance (W/m^2^) under PSC.

PV module	PSC1	PSC2	PSC3
1	750	250	250
2	250	500	0
3	1000	500	250

### Modified boost converter

**Figure 7:**
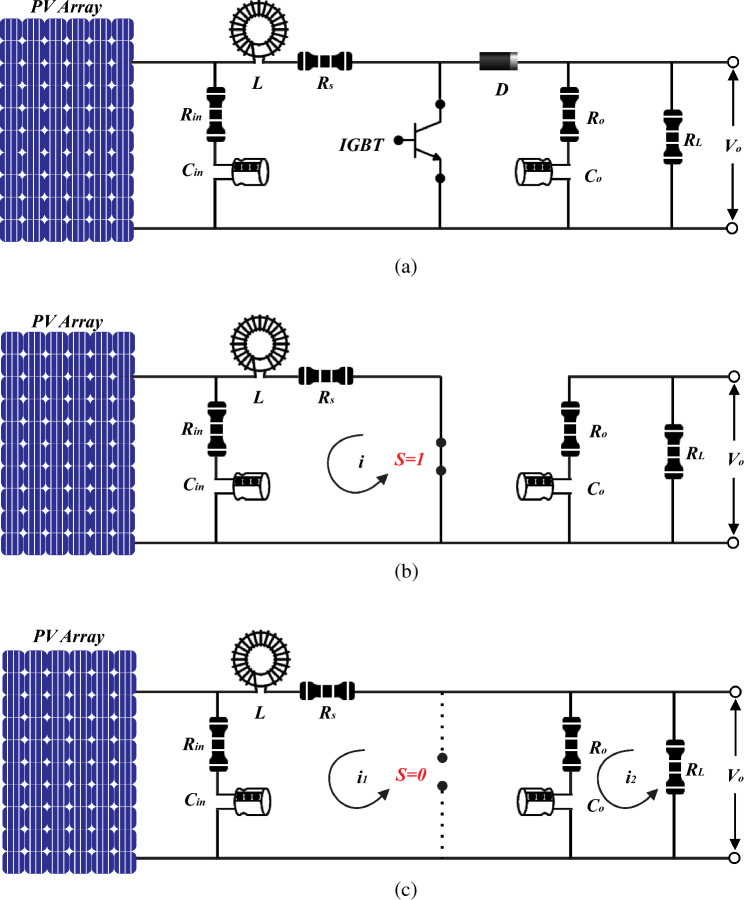
Equivalent model of boost converter (**a**) Schematic circuit diagram (**b**) Switch = ON state (**c**) Switch = OFF.

Commonly, a boost converter is adopted due to its heightened efficiency, it is the preferred choice^14,48^. As depicted in Fig. 7a, the output of the PV array is connected to the modified boost converter. Periodically, the output voltage *v*(*t*) and current *i*(*t*) are sampled at intervals of *Ts* by the controller. Subsequently, the controller calculates the output power *p*(*t*) = *v*(*t*)*i*(*t*) and adjusts the duty cycle *D*(*t*) following the MPC. In this context, the operation is expressed through the Eq. (7).

7 $$D\left[t+1\right]=d\left[t\right]-sgn\left\{\left(p\left[t+1\right]-p\left[t\right]\right)\left(v\left[t+1\right]-v\left[t\right]\right)\right\}\cdot\Delta D$$

where, *y*[t] represents the *‘t’* sample of the waveform *y*(*t*), where t belongs to the set of natural numbers. The function sgn( ) is defined as a modified signum function with sgn(0) equal to + 1. Additionally, Δ*D* > 0 corresponds to the duty cycle increment applied by the APO-MPC controller.

8 $$D=\frac{{V}_{o}}{{V}_{pv}}=\frac{{I}_{pv}}{{I}_{o}}=\frac{1}{1-D}$$

In a boost converter model, input capacitors and resistors are not typically included. Normally, these elements are found on the output side^49^. However, in this study, our model has been extended to incorporate an additional input capacitor and two input resistors.

#### Mode 1

When the switch is in the ON state, as illustrated in Fig. 7b, the inductor *L*, resistor *R*_*in*_, resistor *R*_*s*_, and input capacitor *C*_*in*_ are connected in series with the input voltage *V*_*pv*_. In this scenario, we apply the conventional principles of electricity and magnetism, and according to Kirchhoff's voltage law, we derive the following equation:

9 $$\left\{\begin{array}{c}{V}_{pv} = L\frac{di}{dt}+ I({R}_{in}+{R}_{s})+ V{c}_{in}\\ V{c}_{in} = 1/{C}_{in} \int i dt\\ \frac{di}{dt} =1/L\left[ {V}_{pv}- i\left({R}_{in}+{R}_{s}\right)- 1/{C}_{in} \int i dt\right]\end{array}\right.$$

*Vc*_*in*_ represents the voltage across the input capacitor.

#### Mode 2

When the switch is in the OFF state, as illustrated in Fig. 7c, the inductor *L*, resistor *R*_*in*_, resistor *R*_*s*_, resistor *R*_*o*_, input capacitor *C*_*in*_, and load capacitor *C*_*o*_ are connected in series with the input voltage *V*_*pv*_. Under this condition, the equation following Kirchhoff's voltage law is as follows:

10 $$\left\{\begin{array}{c}\frac{di}{dt}=1/L\left[{V}_{pv}- {i}_{1}\left({R}_{in}+{R}_{s}+{R}_{o}\right)- V{c}_{in}- V{c}_{o}\right]\\ V{c}_{o}=-{i}_{2}({R}_{o}+{R}_{L})\end{array}\right.$$

The switching frequency can be computed by employing the following Equation

11 $${f}_{s}=\frac{1}{T}$$

The internal resistance of the PV array changes in response to the solar irradiance level. It can be determined at 1000 W/m^2^ and 50 W/m^2^, respectively, by applying Eq. (12).

12 $${R}_{IR}=\frac{{V}_{mp}}{{I}_{mp}}$$

Similarly, the output resistance at the output side of the boost converter can be computed using Eq. (13).

13 $${R}_{o}= 2.5*{R}_{IR}$$

At a minimum irradiance of 50 W/m^2^, *V*^*o*^_*mp*_ is measured as ± *10%* of *V*_*mp*_ at STC. Similarly,* P*^o^_*mp*_ is computed as ± *5%* of *P*_*mp*_ at STC*,* then *I*^*o*^_*mp*_ = *P*^*o*^_*mp*_/*V*^*o*^_*mp*_*.*

Computation of duty ratio is given by

14 $${D}_{mp}=1-\sqrt{\frac{{R}_{mp}}{{R}_{o}}}$$

The calculation of the output voltage and current at both 1000 W/m^2^ and 50 W/m^2^ by applying Eq. (15).

15 $$\left\{\begin{array}{c}{V}_{o}=\frac{{V}_{pv}}{1-D}\\ {I}_{o}=\frac{{V}_{o}}{{R}_{o}}\end{array}\right.$$

The ripple values of voltage and current are considered with factors of 0.002 and 0.4 relative to the maximum voltage and current, respectively. The main role of the inductor is to maintain a continuous and consistent output. Therefore, it's crucial to carefully weigh the cost and value of the inductor during the boost converter modeling process. Selecting the right inductor value is essential to achieve a balance between cost-effectiveness and a reliable power supply.

The Eq. (15) can be used to compute the optimal inductor value:

16 $$L=\frac{{V}_{mp}*D}{2{I}_{L}*{f}_{s}}$$

The parameters *R*_*pv*_*, R*_*L*_*, R*_*s*_*, C*_*pv*_*, and C*_*L*_*,* of the boost converter are obtained using the following relations:

17 $$\left\{\begin{array}{c}{R}_{pv,L}= {R}_{IR}(1-{D}^{2})\\ {R}_{s}= {R}_{o}(1-{D}^{2})\\ \genfrac{}{}{0pt}{}{{C}_{pv}=\frac{4{V}_{mp}*D}{\Delta {V}_{pv}*{R}_{pv}*{f}_{s}}}{{C}_{L}=\frac{2{V}_{L}*D}{\Delta {V}_{L}*{R}_{L}*{f}_{s}}}\end{array}\right.$$

## MPPT implementation

**Figure 8:**
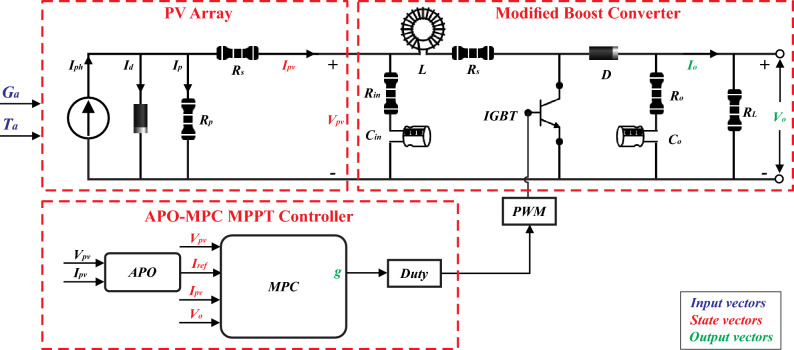
Schematic circuit and control scheme of proposed APO-MPC MPPT algorithm.

Efficiently monitoring the GMPP is crucial for the effective utilization of the PV power generation system. In regions prone to cloudy weather, solar panels frequently encounter partial shading from dust, clouds, and nearby structures. Therefore, deploying a highly effective MPPT system becomes essential to enhance economic efficiency across diverse operational scenarios. The proposed MPPT method combines the APO technique with the adapted MPC algorithm, referred to as APO-MPC. In this approach, APO computes the reference current using appropriate VSS while MPC triggers the converter for optimum tracking of GMPP. The schematic diagram of the proposed algorithm is presented in Fig. 8.

This approach ensures prompt and rapid tracking under PSCs. As a result, it enhances tracking speed without any computational complexity or compromising the DDCC.

### APO algorithm

**Figure 9:**
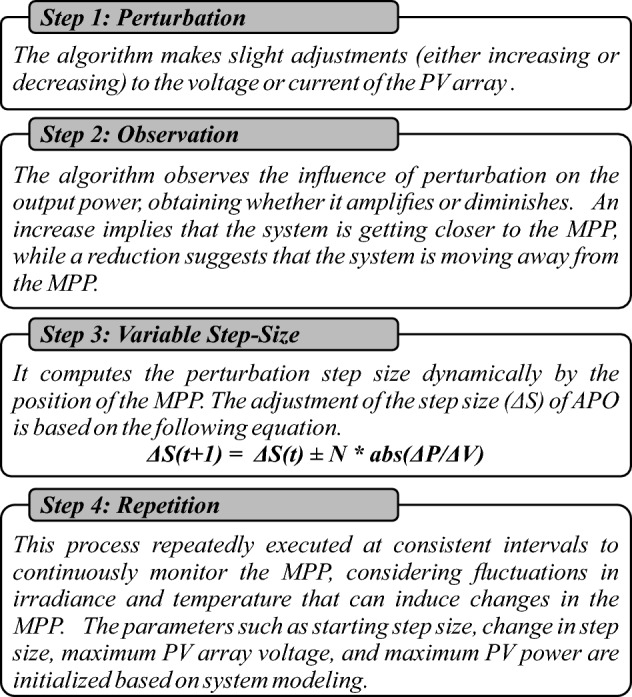
The operational framework of APO.

The traditional P&O controller perturbs the voltage of the array by a constant small increment or decrement and adjusts the output power. If the power increases, the controller continues to adjust in the same direction until the power no longer increases. However, due to constant increments and decrements, lead to oscillations in output power. It lacks the capability to monitor power fluctuations during load modifications and quick changes in environmental conditions. The operational framework of APO algorithm is presented in Fig. 9.

**Figure 10:**
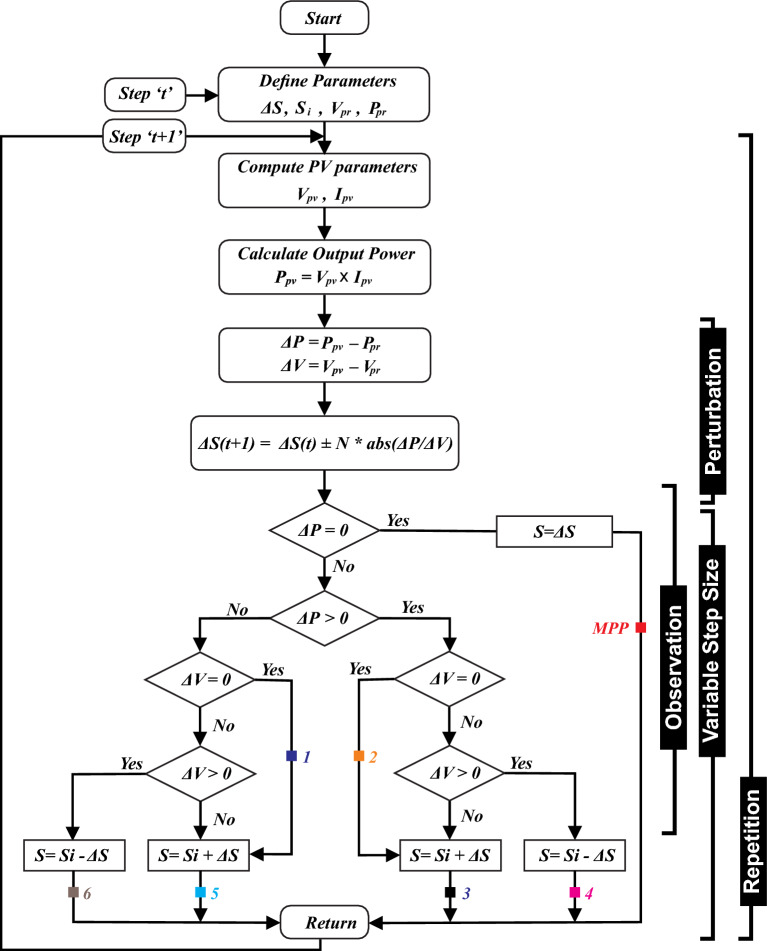
Flowchart of APO algorithm including each operational step.

**Figure 11:**
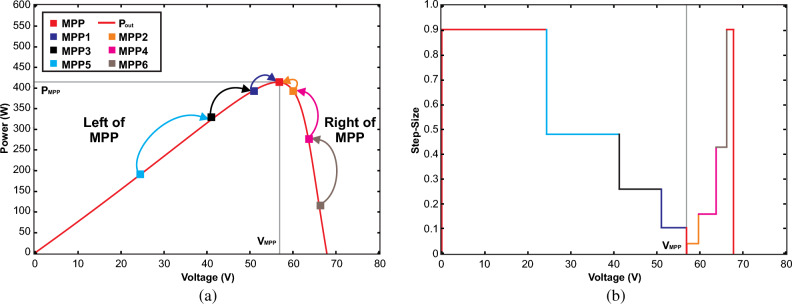
At different operating points (**a**) MPP tracking trajectory (**b**) Computation of VSS.

APO is an enhanced version of the P&O as presented in Fig. 10. The algorithm functions by systematically altering the operating point of the PV system and observing the consequent variations in power production until the MPP is attained, as depicted in Fig. 11a. It declares VSS according to the operating point of MPP with a scaling factor *N*. The main goal of the APO algorithm is to increase the speed at which the PV system tracks the MPP and reduce oscillations around the MPP. This leads to an improvement in the overall efficiency of the PV system.

18 $$\Delta S\left(t+1\right)=\Delta S\left(t\right)\pm N*abs\left(\frac{\Delta P}{\Delta V}\right)$$

The algorithm operates in four steps i.e. perturbing the voltage or current of PV array, observing the impact of perturbation, computing VSS dynamically, and repeating the process continuously for consistent intervals as depicted in Fig. 9.

Figure 11a illustrates the detection method and the step size for each cycle of operation. Initially, when the algorithm starts, it sets the operating voltage in an open-circuit state. This involves progressively integrating the value of $\Delta S(t)$ into Eq. (15), which results in a consecutive sequence of operating points for the PV array. The path followed and the corresponding step sizes are depicted in Fig. 11b. Additionally, when the difference between consecutive operating points becomes 0, the sequence of operations will converge toward the MPP through a specific process, and the step size will simultaneously reduce to zero.

### Proposed hybrid APO-MPC algorithm

MPC stands as an advanced control methodology applicable for regulating a wide range of systems. Usually, it functions by employing a system model to predict its future actions. This model is utilized to calculate a control input, striving to minimize a cost function that typically signifies the difference between the system's output and a desired reference. Then, the computed control input is applied to the system, initiating a repetition of the entire process.

**Figure 12:**
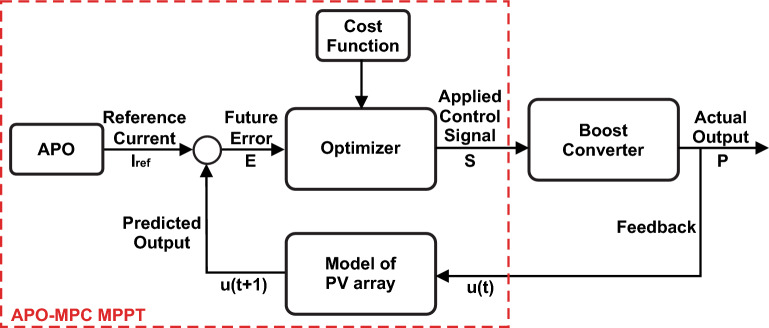
Operational block diagram of MPC.

The proposed algorithm demonstrates a high degree of effectiveness in predicting the future behavior of the PV system. It achieves this by evaluating various possible control actions within a set time frame. In numerous applications, the load is unpredictable and varies over time. Consequently, it's essential to include an estimation feature to reduce the impact of output voltage discrepancies due to load uncertainties. To address any divergence from the true reference value, the output current's reference is suitably modified, employing the APO-based GMPPT model illustrated in Fig. 12.

The best control action for the upcoming step is selected based on the forecasted future conditions of the system. This selection aims to minimize a particular cost function. The study adopts a one-step prediction method *N* = *1*, where the MPC predicts the system's behavior in the next sampling interval, labeled as *u*(*t* + *N*) with *N* being *1*. Depending on the specific application and required performance, it might be necessary to project further into the future, encompassing more steps. By analyzing the anticipated reaction of the system to control inputs, the optimal next state for switching is identified by minimizing a cost function.

**Figure 13:**
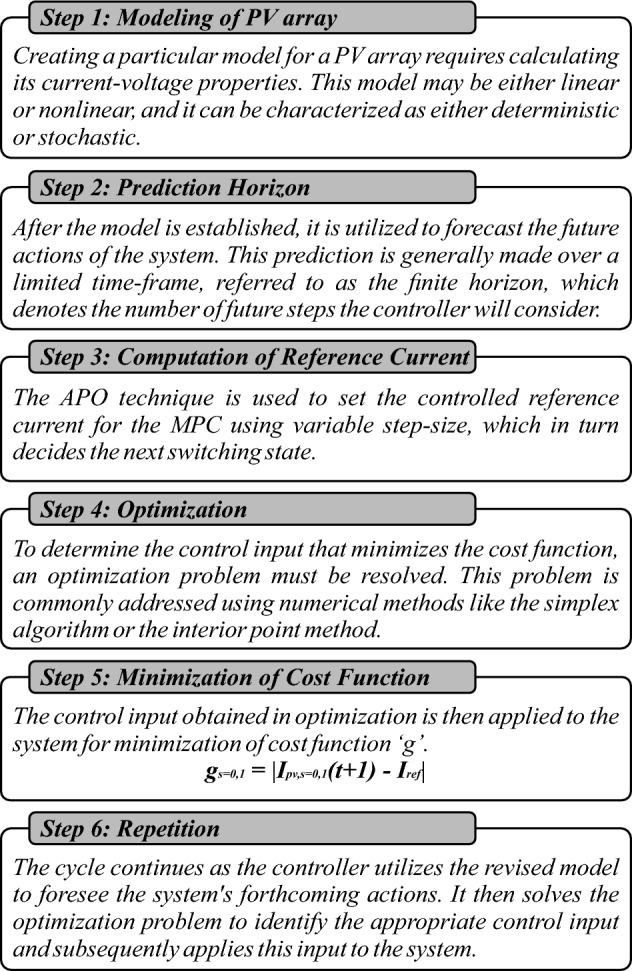
The operational framework of MPC.

The algorithm operates in six steps i.e. mathematical modeling of PV array, forecasting future response in a prediction horizon, computation of reference current through APO, optimizing the process continuously for obtaining the control input, minimization of cost function, and repetition till convergence towards GMPP as depicted in Fig. 13.

**Figure 14:**
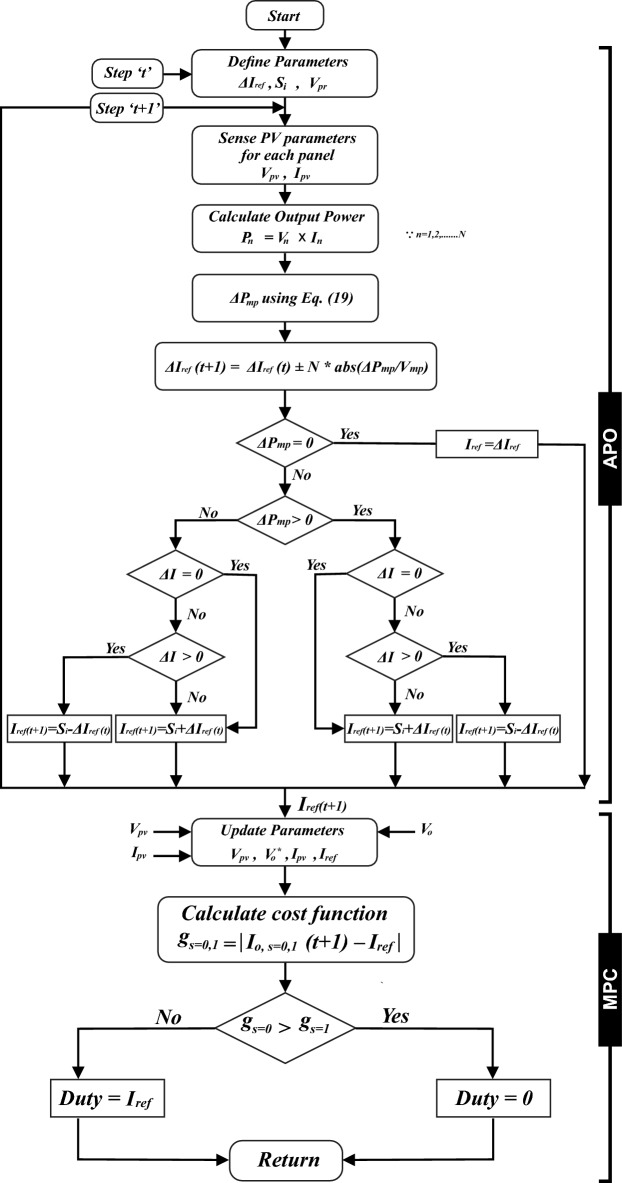
Flowchart of proposed APO-MPC algorithm including each operational step.

First, the algorithm identifies the change in solar irradiance on the basis of change in output power *ΔP*_*mp*_ with each module using Eq. (19). From the change in power or MPP detection against each series module, algorithm identifies the shaded modules to define the number of LMPPs. If the number of shaded modules is M out of number of series connected N modules, the number of LMPPs would be equal to M. Next, it computes the V_mp_ using Eq. (20), for the non-shaded PV modules as voltage across shaded module is V_D_ = 0.7V. Then, APO method calculates the I_ref_ based on desired output power of the whole string using Eq. (21) to be followed by the MPC which in turn identifies the next switching state to optimize output power at GMPP, as presented in Fig. 14. This way, algorithm processes the recurring procedure to avoid any LMPP and modify the value of I_ref_ and V_mp_ accordingly using Eq. (22).

19 $${\Delta P}_{mp}={P}_{a}-{P}_{b}$$

where (a,b) = $$\frac{N!}{2!(N-2)!}$$

20 $${V}_{{mp}_{y}}=81\%*\frac{N-y}{N}{V}_{oc}$$

21 $${I}_{ref}={S}_{i}\pm \Delta {I}_{ref}$$

22 $$\left({I}_{ref},{V}_{mp}\right)=f\left(max\left\{{{P}_{LMPP}}_{y}\right\}\right)$$

where P_LMPP_ is known as output power of LMPP at y-th PV module.

The state-space model is employed as a discrete-time universal format for the non-linear control variables. This model aids in their prediction within the framework of the proposed algorithm. Suppose, a generic system defines *x* as a state vector, and *u* and *y* as input and output vectors respectively.

23 $$\dot{x}\left(t\right)=Ax\left(t\right)+Bu(t)$$

24 $$y\left(t\right)=Cx\left(t\right)$$

**Figure 15:**
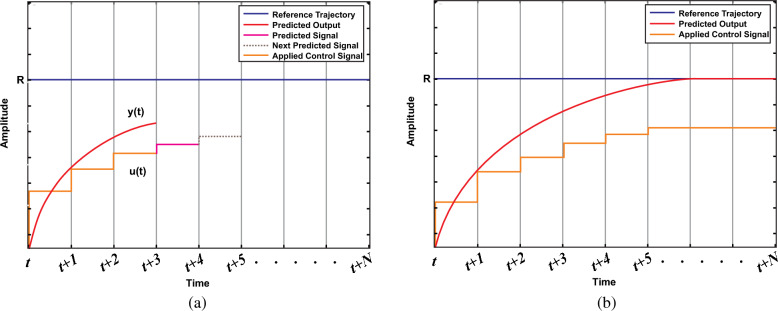
MPC control action under prediction horizon (**a**) at u(t) to u(t + 2) (**b**) at u(t) to u(t + N).

The cost function 'g' is optimized for a specific time step within the time horizon *N*. This optimization yields a set of *N* ideal control actions, from which the controller only uses the first. Accordingly, the initial control signal *u*(*t*) is implemented in the process to follow a reference trajectory *R*, and the rest of the anticipated control signals are disregarded. This method is chosen because the outcome at the next sampling point is already known. Having a greater prediction horizon enables the MPC to predict future behavior effectively, and accommodate forthcoming disturbances in the system's dynamics. On the other hand, the control horizon signifies the number of control steps computed for optimizing the output. A longer control horizon allows the MPC to plan more future states lead better performance, however, it demands more computational power to execute. Figure 15a illustrates the prediction horizon at *u*(*t*), *u*(*t* + *1*), and *u*(*t* + 2), while Fig. 15b shows the prediction horizon from *u*(*t*) to *u*(*t* + *N*)*.* Equations (25–27) represent 4 state vectors, 2 input vectors, and 3 output vectors as presented in Fig. 8.

25 $$x=\left[{V}_{pv}, {I}_{pv},{I}_{ref}, {V}_{o}\right]$$

26 $$u=\left[{G}_{a}, {T}_{a}\right]$$

27 $$y=\left[{g, V}_{o}, {I}_{o}\right]$$

This technique predicts the error at the upcoming sampling time and optimizes the cost function 'g' to ascertain the switching state. The predictive controller considers inputs like the current and voltage of the PV system and the controlled reference current. Through a series of discrete-time equations, the future behavior of the control variable at the next sampling time (t + 1) is forecasted.

When operating in continuous conduction mode, the discrete-time equations that describe the boost converter's behavior, as illustrated in Fig. 7b,c, are represented by Eqs. (28) and (29) for the "ON" and "OFF" states of the switch. The components *R*_*in*_, *R*_*s*_, and *C*_*in*_ are disregarded in these equations because their values are extremely small, simplifying the system's computational complexity.

28 $$\frac{d{I}_{o}}{dt}={I}_{pv}-\left(1-S\right)\frac{1}{L}{V}_{{c}_{o}}+\frac{1}{L}{V}_{pv}$$

29 $$\frac{d{V}_{{c}_{o}}}{dt}=\left(1-S\right)\frac{1}{{C}_{o}}{I}_{o}+\frac{1}{{R}_{o}{C}_{o}}{V}_{{c}_{o}}$$

To ascertain the pulse *S* state in the MPPT controller, the cost function g is minimized by adhering to the steps depicted in Fig. 14. When *S* equals 0, Eqs. (28) and (29) are reformulated as follows:

30 $${I}_{o,s=0}(t+1)={I}_{pv}-\frac{{T}_{s}}{L}\left({V}_{{c}_{o}}+{V}_{pv}\right)$$

31 $${V}_{{c}_{o,s=0}}\left(t+1\right)=\frac{{T}_{s}}{{C}_{o}}\left({I}_{o}-\frac{{V}_{{c}_{o}}}{{R}_{o}}\right)$$

In the previously mentioned equations, 'Ts' represents the sampling time. When the number of steps rises to two or three, an increase in computation time naturally follows. However, this exchange leads to enhanced control performance. Likewise, if S equals 1, Eqs. (28) and (29) can be reformulated as follows:

32 $${I}_{o,s=1}(t+1)={I}_{pv}+{T}_{s}\frac{{V}_{pv}}{L}$$

33 $${V}_{{c}_{o},s=1}\left(t+1\right)=\frac{{T}_{s}}{{R}_{o}{C}_{o}}{V}_{{c}_{o}}$$

The control input that minimizes the cost function is determined by solving an optimization problem. Typically, this problem is addressed using a numerical method stated in Eq. (34).

34 $$\underset{s}{{\text{min}}}g(s=1 or 0)$$

The cost function is constructed by considering the future states, references, and expected control actions as follows:

35 $$g=f(x\left(t\right),u\left(t\right),\dots ,u\left(t+N-1\right))$$

The cost function can be represented by the following equation.

36 $${g}_{s=\mathrm{0,1}}=\left|{I}_{o,s=\mathrm{0,1}}(t+1)-{I}_{ref}\right|$$

Finally, it obtains the real-time feedback from output side sensor Vo to verify whether the system is operating at the GMPP by continuously updating its predictions and control actions.

## Results and discussions

**Figure 16:**
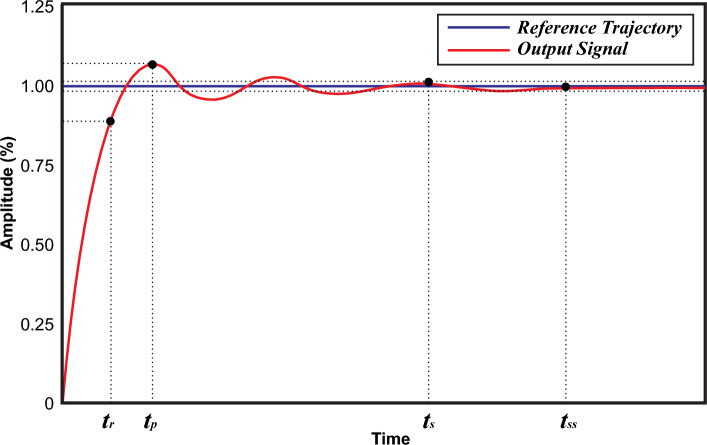
Transient and steady-state response time of an output signal.

**Figure 17:**
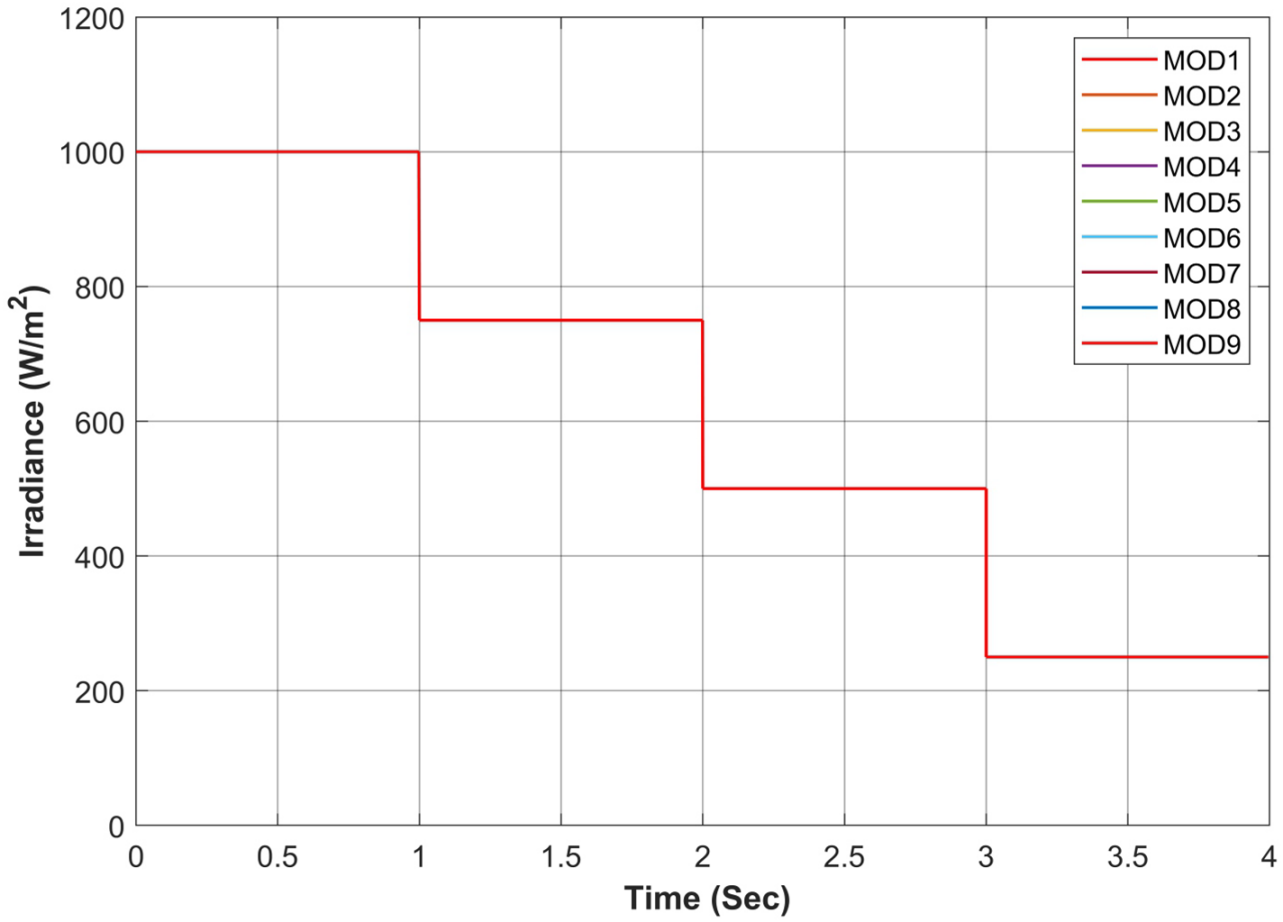
Simultaneous constant solar irradiance on each module.

**Figure 18:**
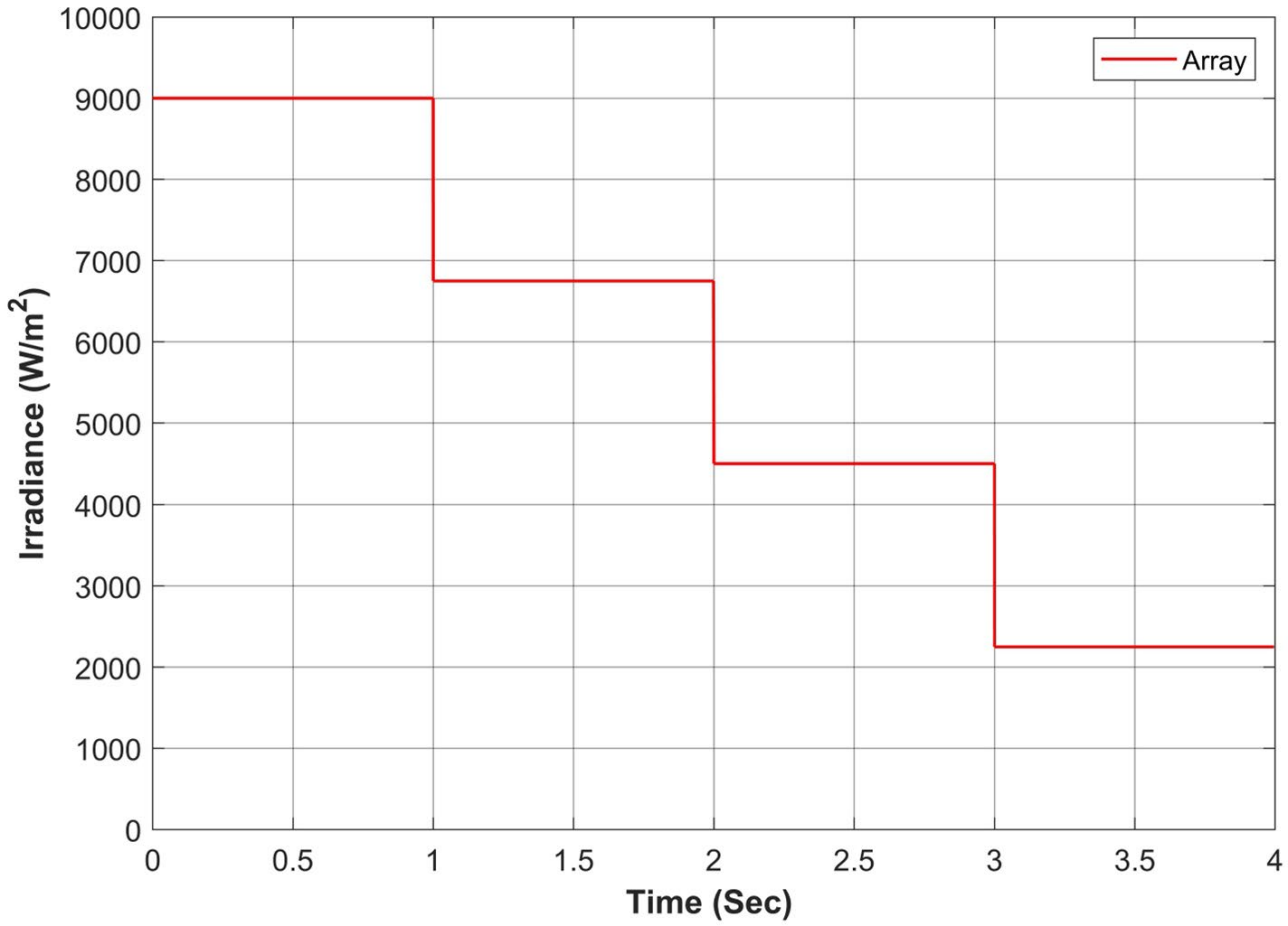
Cumulative solar irradiance under constant irradiance profiles on PV array.

**Figure 19:**
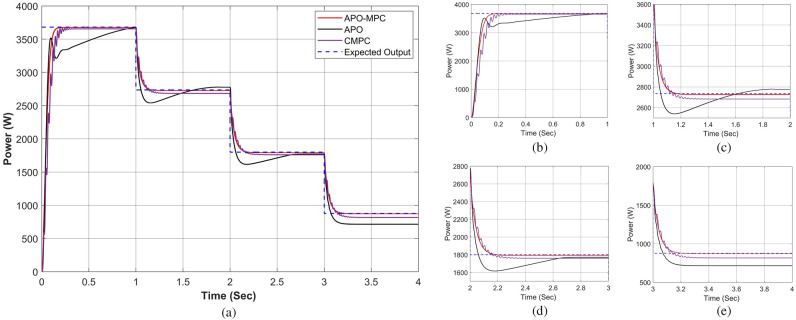
Performance comparison of proposed approach under constant solar irradiance (**a**) output power for complete horizon t = 0–4s (**b**) output behavior in segment 0–1s (**c**) output behavior in segment 1–2s (**d**) output behavior in segment 2–3s (**e**) output behavior in segment 3–4s.

**Figure 20:**
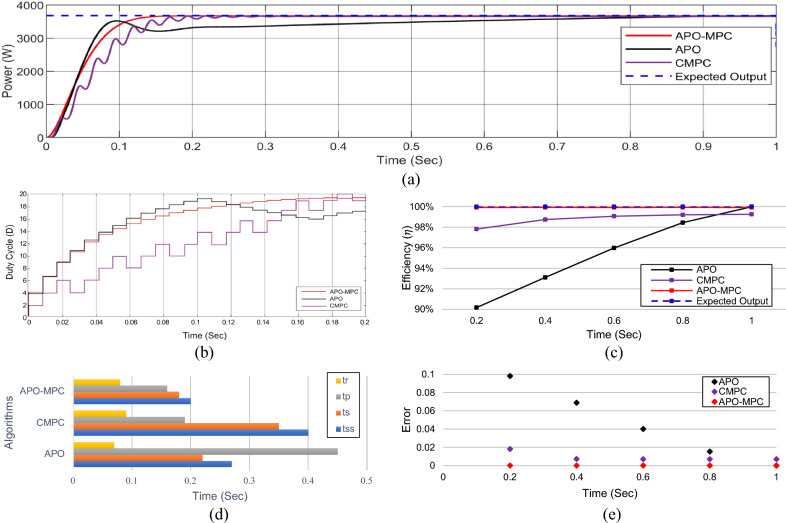
Simulation experiment results of case I under constant irradiance profiles (**a**) PV output power for t = 0–1s (**b**) duty cycle for t = 0–0.2s (**c**) comparison of GMPP tracking efficiency for t = 0–1s (**d**) comparison of step response for t = 0–0.5s (**e**) error analysis of proposed algorithm with APO and CMPC for t = 0–1s. The average tracking efficiencies of all algorithms were obtained as (APO-95.54%), (CMPC-98.82%) and (APO-MPC 99.93%).

**Figure 21:**
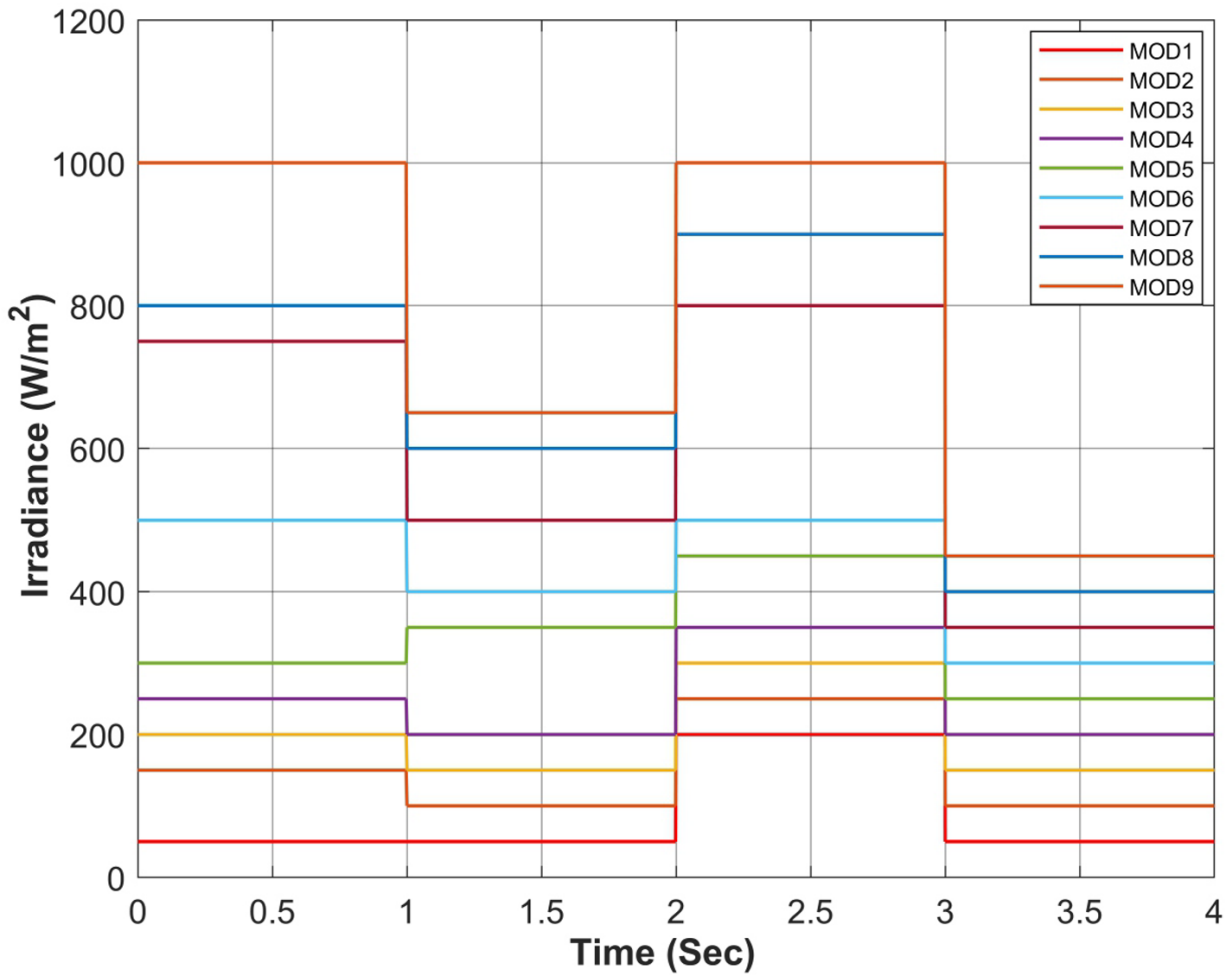
Rapidly changing solar irradiance on each module.

**Figure 22:**
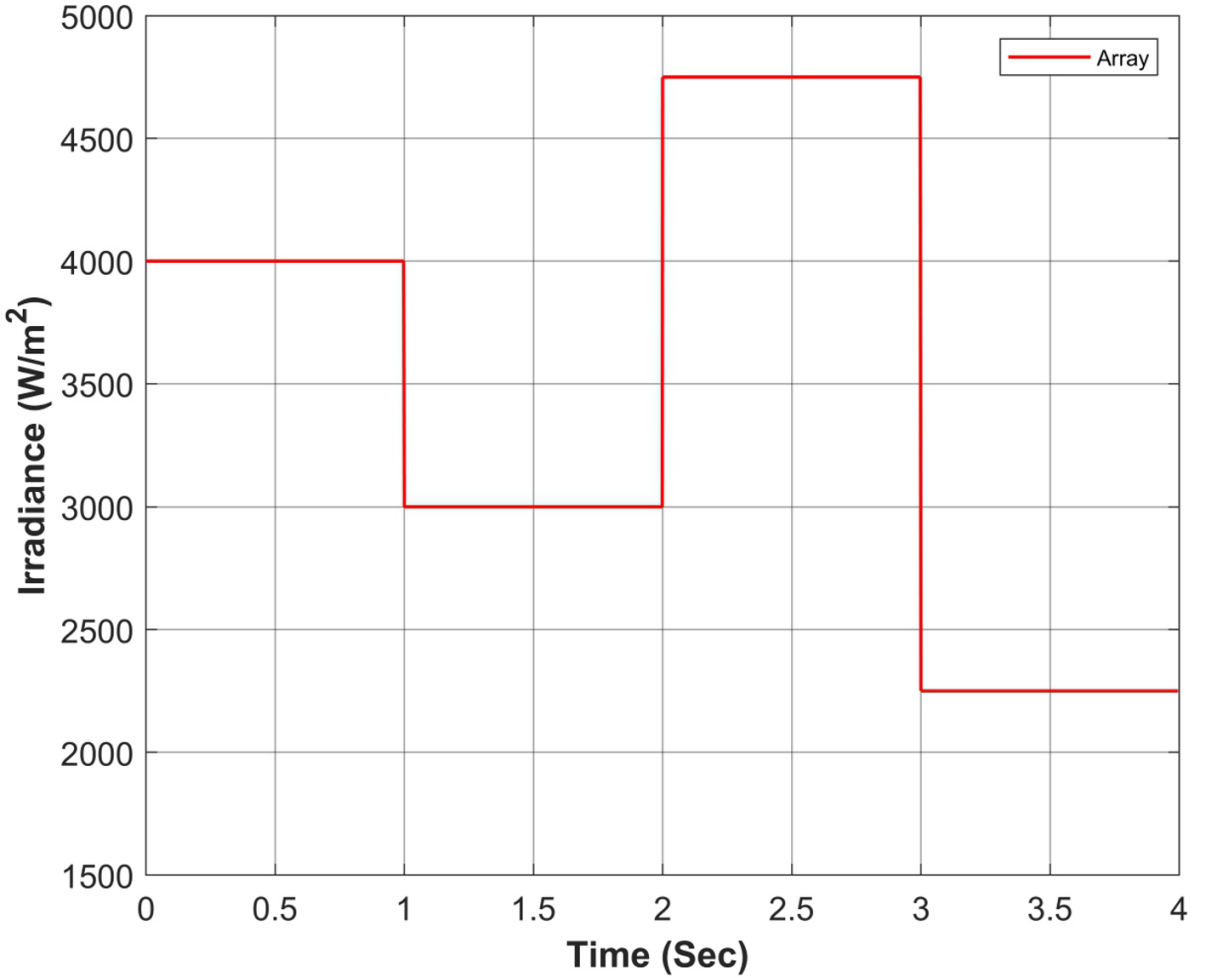
Cumulative solar irradiance under rapidly changing profiles on PV array.

**Figure 23:**
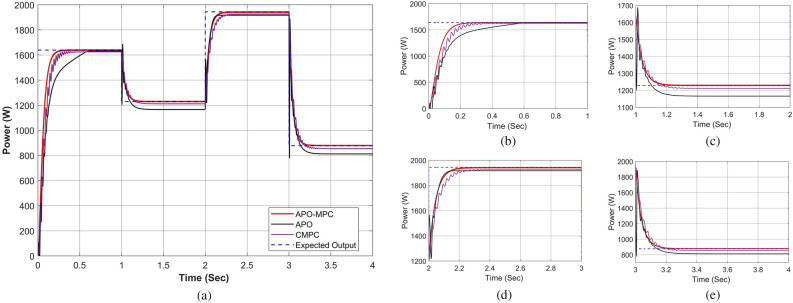
Performance comparison of proposed approach under rapidly changing solar irradiance (**a**) output power for complete horizon t = 0–4s (**b**) output behavior in segment 0–1s (**c**) output behavior in segment 1–2s (**d**) output behavior in segment 2–3 (**e**) output behavior in segment 3–4s.

**Figure 24:**
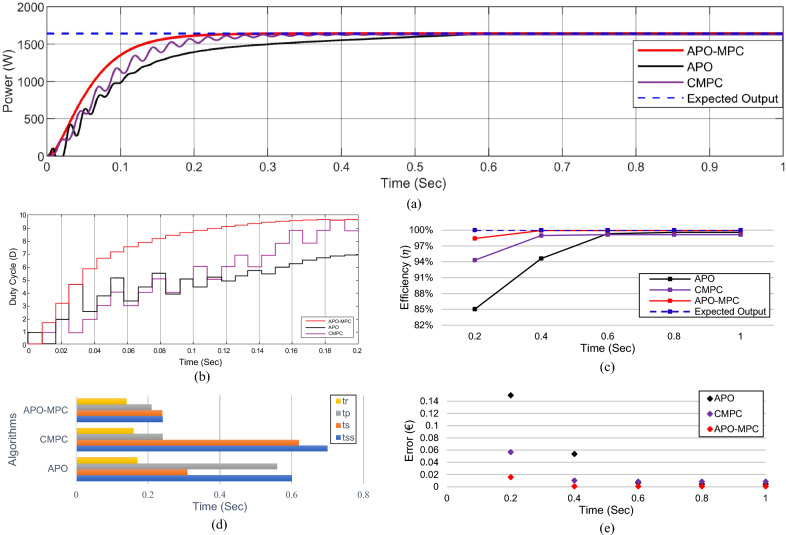
Simulation experiment results of case II under rapidly changing irradiance profiles (**a**) PV output power for t = 0–1s (**b**) duty cycle for t = 0–0.2s (**c**) comparison of GMPP tracking efficiency for t = 0–1s (**d**) comparison of step response for t = 0–0.8s (**d**) error analysis of proposed algorithm with APO and MPC for t = 0–1s. The average tracking efficiencies of all algorithms were obtained as (APO-95.63%), (MPC-98.14%) and (APO-MPC 99.63%).

**Figure 25:**
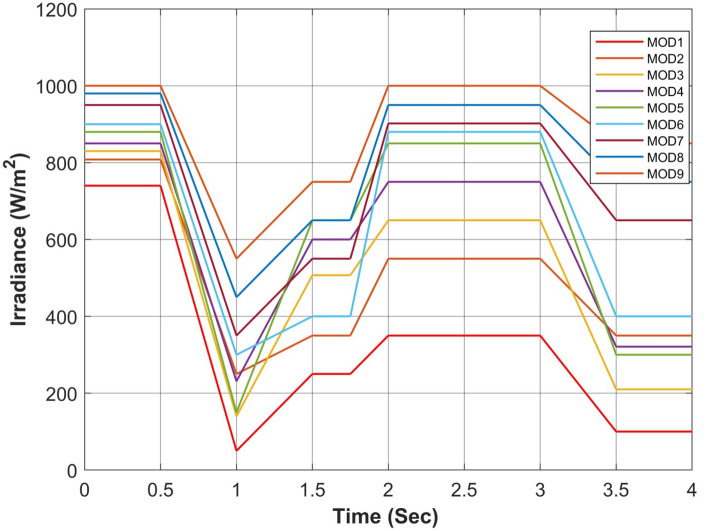
Simultaneous rapidly changing solar irradiance on each module.

**Figure 26:**
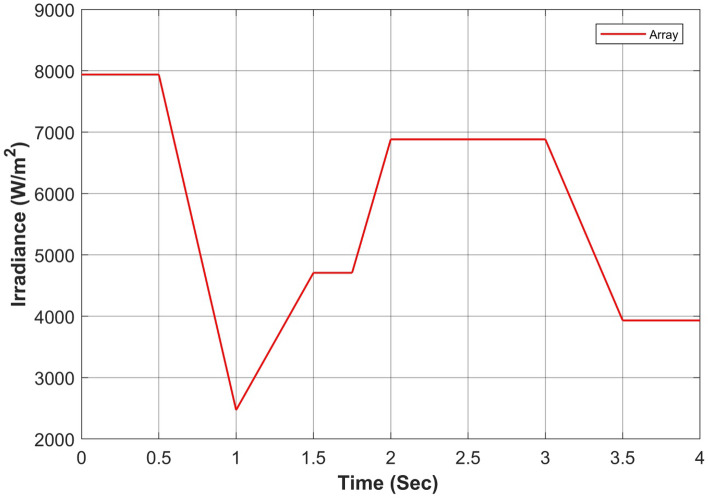
Cumulative solar irradiance under linearly changing profiles on PV array.

**Figure 27:**
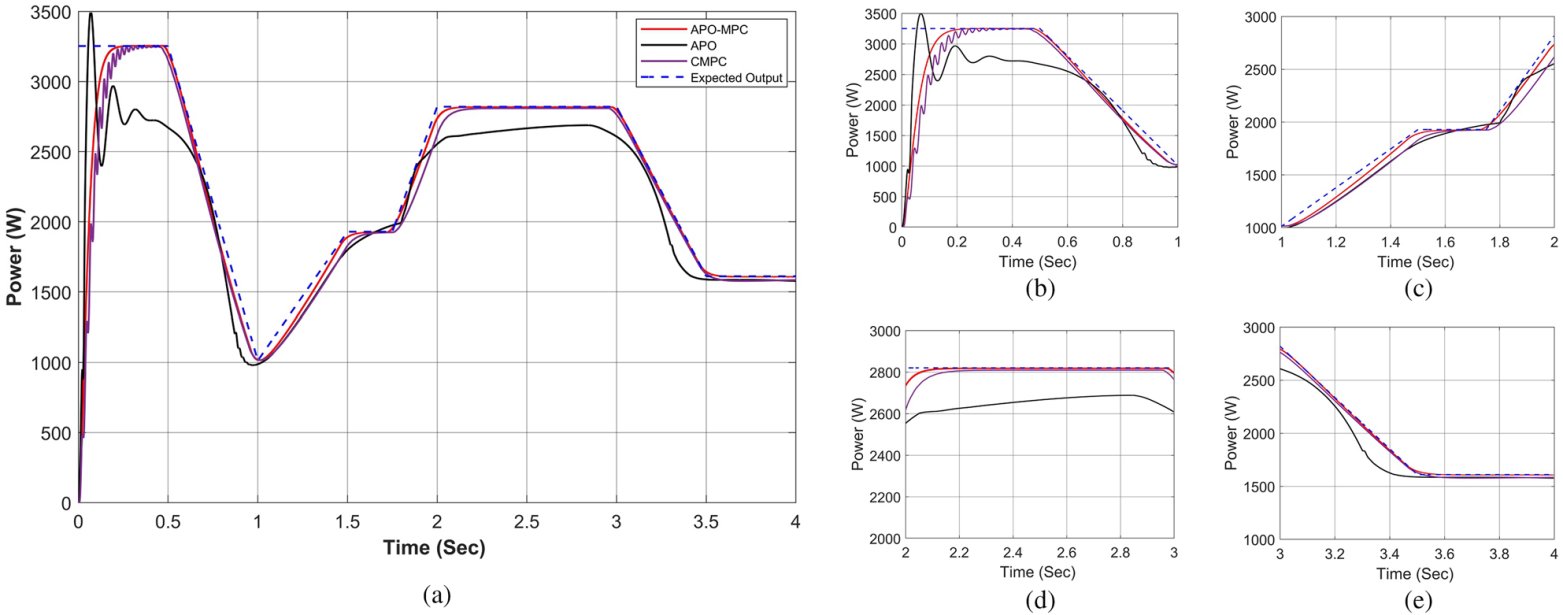
Performance comparison of proposed approach under linearly changing solar irradiance (**a**) output power for complete horizon t = 0–4s (**b**) output behavior in segment 0–1s (**c**) output behavior in segment 1–2s (**d**) output behavior in segment 2–3 (**e**) output behavior in segment 3–4s.

**Figure 28:**
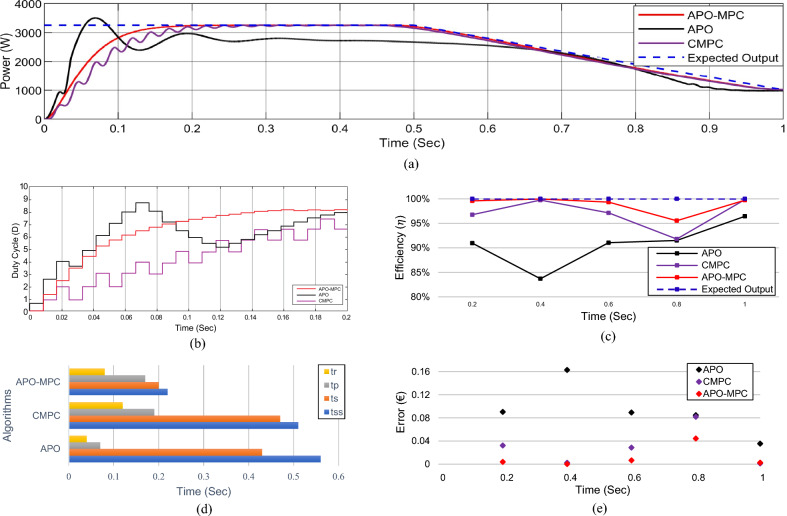
(**a**) Simulation experiment results of case III under linearly changing irradiance profiles (**a**) PV output power for t = 0–1s (**b**) duty cycle for t = 0–0.2s (**c**) comparison of GMPP tracking efficiency for t = 0–1s (**d**) comparison of step response for t = 0–0.7s (**d**) error analysis of proposed algorithm with APO and CMPC for t = 0–1s. The average tracking efficiencies of all algorithms were obtained as (APO-81.42%), (CMPC-93.62%) and (APO-MPC 94.67%).

This paper aims to develop a high-performance tracker to enhance the overall effectiveness of PV systems across various climatic scenarios. The suggested algorithm is modeled through simulations using the MATLAB Simulink R2023a tool. The performance of a PV system is directly influenced by the level of irradiance it encounters. In real-world scenarios, irradiance levels can change swiftly, leading to substantial variations in the output power of modules when there are extreme shifts in irradiation. Therefore, assessing the efficiency of MPPT under varying irradiance conditions becomes a vital task. In this configuration, nine individual PV modules are interconnected in series and parallel combinations to form the PV array. To ensure timely acquisition of MPP readings once the system reaches a steady-state condition, the sampling time for the DC-DC boost converter MPPT algorithm is set to 0.02 s. This choice accounts for the transient response of MPPT inputs, such as PV voltage and PV current, preventing delays in tracking maximum power and avoiding system failure. The simulations presented in Figs. 16, 17, 18, 19, 20, 21, 22, 23, 24, 25, 26, 27, 28 illustrate the testing of the PV system under various PSCs.

The following discussion involves a simulated model of the modified boost converter using its equivalent model outlined in Eq. (3). As explained previously, this method is chosen to enable the direct specification of the duty cycle ‘D’ as a simulation parameter. The simulation focuses on the array consisting of 9 PV modules, and its relevant parameter details and characteristics are presented in Table 3. The modeled values for the modified boost converter components are outlined in Table 5. Given that the objective of employing APO-MPC is to achieve rapid tracking through APO and utilize MPC for precise duty cycle adjustments, we choose ${f}_{s}$ of 5kHz to facilitate this goal. Table 6 illustrates the output power rating of each PV module.

**Table 5:** Modeled parameters of boost converter under PSC.

Parameter	Symbol	Value
Source resistor	*R*_*s*_	100 mΩ
Load resistor	*R*_*L*_	50 mΩ
Input resistor	*R*_*in*_	10 mΩ
Input capacitance	*C*_*in*_	0.1 mF
Output capacitance	*C*_*o*_	4700 µF
Output resistor	*R*_*o*_	21.5 Ω
Inductance	*L*	100 mH
Input voltage ripple	*ΔV*_*pv*_	0.129 V
Output voltage ripple	*ΔV*_*o*_	1.71 V
Switching frequency	*f*_*s*_	5 kHz
Sampling time	*T*_*S*_	20 ms
Current ripple	*ΔI*	91 mA

**Table 6:** Output power rating of each PV module.

Irradiance (W/m^2^)	Power rating
1000	409.82 W
750	304.71 W
500	200.35 W
250	97.52 W

### Performance evaluation

The evaluation of the MPPT methods' effectiveness relies on the assessment of the following three criteria:

***Tracking efficiency***

This criterion evaluates the overall efficiency of GMPP tracking by using Eq. (37).

37 $$\eta = \frac{{P}_{a}}{{P}_{GMPP}}\times 100$$

where $\eta$ is the tracking efficiency, *P*_*a*_ is the actual output power of the PV system at any given time, *P*_*GMPP*_ is the maximum power that the PV system could produce under PSCs.

#### Transient response time (T.R)

It is a measure of how quickly the MPPT algorithm can adapt to changes in shading conditions and bring the PV system back to its MPP after a disturbance. This response time is important because it affects the system's ability to capture the available solar energy efficiently. The rise time ‘*t*_*r*_’ is the time it takes for the system’s transition from 10 to 90% of its ultimate or steady-state value is known as the rise time. Similarly, peak time ‘*t*_*p*_’ is the duration it takes for the system to attain its highest overshoot.

#### Steady state response (S.R)

It refers to how well the system maintains operation at or near the MPP after it has adapted to changing shading conditions. Achieving a steady-state response is important because it ensures that the system consistently operates at its maximum output power despite changes in partial shading. The time required for attaining this state is denoted by ‘*t*_*ss*_’. Settling time ‘*t*_*s*_’ is the duration it takes for the transient response to enter and remain within a range of ± 2% of the final or steady-state value. Figure 16 presents both T.R and S.R of an output signal.

### Performance validation

The performance of the proposed algorithm has been validated by employing two control algorithms in comparison with the proposed APO-MPC in the context of PSCs. APO operates by assessing changes in both the voltage and current of the PV array to ascertain if the operating point aligns with the MPP on the P–V characteristic curve. However, in PSCs, APO may lead to be settled into the LMPP. Conversely, CMPC MPPT algorithms utilize optimization techniques to iteratively search for the GMPP. However, the oscillations in output power are prolonged due to the continuous need for measurement and iteration in the pursuit of optimization processes. The designed and modeled parameters of the PV array and boost converter are presented in Tables 3 and5 respectively.

#### Case I: constant irradiance profiles

The proposed algorithm has been verified through simulations conducted under constant solar irradiance conditions with PSC scenarios on each module ranging from 250 to 1000 W/m^2^, as depicted in Table 7. The irradiance changes simultaneously on each module with the instant of 1 s. Figures 17 and 18 depict simultaneous irradiance on each module and cumulative irradiance on the array respectively. Under constant irradiance profiles, the output power behavior is validated on a patch of 1 s over a total time horizon of 4 s. The duty cycle of the modified boost converter is adjusted by the PWM signal generated from MPC to change the optimal point of the PV array. The ratings of output power patterns are illustrated in Table 8.

**Table 7:** Constant solar irradiance (W/m^2^) on each module.

PV module	t = 0−1s	t = 1−2s	t = 2−3s	t = 3−4s
MOD1	1000	750	500	250
MOD2	1000	750	500	250
MOD3	1000	750	500	250
MOD4	1000	750	500	250
MOD5	1000	750	500	250
MOD6	1000	750	500	250
MOD7	1000	750	500	250
MOD8	1000	750	500	250
MOD9	1000	750	500	250
Array	9000	6750	4500	2250

**Table 8:** Output power rating of 3 × 3 PV array under constant irradiance.

Irradiance (W/m^2^)	Power rating
9000	3688.36 W
6750	2742.39 W
4500	1803.15 W
2250	877.68 W

As the operating point is far away, APO has a larger step during transient response which resulted overshoot in output power and then it reduced the step size which caused a slow tracking response as seen in Fig. 19a. Due to simultaneous irradiance on each module in this scenario, there is only one peak to track by the algorithms.

The expected output power of the PV system is computed as 3688.36W. APO presented an overshoot of 3520.47W during transient response and then it achieved GMPP by linearly rising at 1 s with oscillation of ± 4W during steady-state response. The CMPC algorithm expressed fluctuations during transient response due to rising and falling steps of the duty cycle as presented in Fig. 20b and achieved 3655.41W output power without oscillations in steady-state. However, it reached to steady state with a slight delay of 0.47s. Figure 19b illustrates that the APO-MPC can achieve GMPP at 0.12s and stably operate at 0.13s; the CMPC can achieve GMPP at 0.17s and stably operate at 0.34s; the APO has achieved GMPP at 0.89s Considering the output power at t = 0–1s as shown in Fig. 20a, the duty cycle of APO varies from larger to smaller steps causing the overshoot and also not converging towards GMPP. The CMPC tries to bound the step of the duty cycle to prevent the output from overshooting but it causes many fluctuations while achieving the GMPP. Finally, the APO-MPC achieved GMPP very smoothly without oscillations with respect to APO and CMPC. Figures 20c,e present a comparison of overall efficiency and error analysis in terms of deviation from a reference trajectory. Figure 20d illustrates the step response of all algorithms, from which, APO-MPC has reduced tracking time. The proposed algorithm achieved GMPP precisely by runtime computations of *I*_*ref*_ and optimizing the operating point with an overall efficiency of 99.93%. The proposed algorithm demonstrates efficient tracking of the GMPP across constant weather conditions, including instances of partial shading. This leads to an enhanced overall efficiency of the PV system. Simulation and implementation results indicate that the suggested algorithm holds promise as a solution to enhance the economic performance of PV installations in sandy climatic profiles.

#### Case II: rapidly changing profiles

The validation of the proposed algorithm has been conducted under PSC with rapidly changing solar irradiance profiles on each module as illustrated in Table 9. The irradiance changes rapidly on each module with the instant of 1 s. In this scenario, there is an abrupt shift in solar radiation levels at t = {1,2,3} seconds. Yet, in common real-world situations, solar radiation experiences gradual fluctuations attributed to factors such as passing clouds or the buildup of sand or dust particles. Figures 21 and 22 depict rapidly changing irradiance on each module and cumulative irradiance on the array respectively. The ratings of the output power pattern are illustrated in Table 10.

**Table 9:** Rapidly changing solar irradiance (W/m^2^) on each module.

PV module	t = 0−1s	t = 1−2s	t = 2−3s	t = 3−4s
MOD1	50	50	200	50
MOD2	150	100	250	100
MOD3	200	150	300	150
MOD4	250	200	350	200
MOD5	300	350	450	250
MOD6	500	400	500	300
MOD7	750	500	800	350
MOD8	800	600	900	400
MOD9	1000	650	1000	450
Array	4000	3000	4750	2250

**Table 10:** Output power rating of 3 × 3 PV array under rapidly changing irradiance profiles.

Irradiance (W/m^2^)	Power rating
4000	1639.28 W
3000	1229.46 W
4750	1943.99 W
2250	877.68 W

At the start the operating point is not too far from GMPP, so APO did not result in the overshoot due to moderate steps during transient response as illustrated in Fig. 23a. However, due to small step sizes, it expressed a slow tracking response as seen in Fig. 23b. The expected output power of the PV system is computed as 1639.28W. APO achieved an output power of 1632.14W by a nearly linear rise from 0.21–0.57 at 0.64 s with oscillation of ± 1W during steady-state response.

The CMPC algorithm expressed fluctuations while achieving the GMPP due to unit change in rising and falling steps of the duty cycle as presented in Fig. 24b and achieved 1625.19W output power with oscillations of ± 2W in steady-state at 0.81s. The proposed algorithm illustrated smooth transient response by achieving 1638.25W output power at 0.32 s without oscillations during steady-state response. In this case, the response time of the proposed algorithm is also faster than other algorithms. While rapid fall in irradiance, only APO-MPC followed expected output trajectory at 1230.91W accurately as presented in Fig. 23c. Finally, we can observe in Fig. 23d,e that the proposed algorithm expressed more accurate and efficient behavior nearer to GMPP.

Considering the output power at t = 0–1s as shown in Fig. 24a, the reference operating point is not far due to low irradiance on each module, therefore, APO causes slow convergence towards GMPP. The CMPC tries to achieve the operating point sharply but causes many fluctuations. Finally, the APO-MPC achieved GMPP very smoothly without oscillations with respect to APO and CMPC. Figures 24c,e present a comparison of overall efficiency and error analysis in terms of deviation from the reference operating point respectively. Figure 24d illustrates the step response of all algorithms, from which, APO-MPC has minimum GMPP tracking time over other algorithms.

The proposed algorithm achieved GMPP precisely by runtime computations of *I*_*ref*_ and optimizing the operating point with an overall efficiency of 99.63%. The proposed algorithm demonstrates efficient tracking of the GMPP across rapidly changing weather conditions under PSCs. Simulation and implementation results indicate that the proposed algorithm holds promise as a solution to enhance the economic performance of PV installations in partially shaded locations.

#### Case III: linearly changing profiles

Finally, the validation of the proposed algorithm has been conducted across linearly changing irradiance profiles under PSC on each module as illustrated in Table 11. The irradiance changes gradually on each module with the instant of each sec. In this case, there is a gradual change in solar radiation levels at t = {1,2,3} seconds. Figures 25 and 26 depict linearly changing irradiance on each module and cumulative irradiance on the array respectively. The ratings of the output power pattern are illustrated in Table 12.

**Table 11:** Linearly changing solar irradiance (W/m^2^) on each module.

PV module	t = 0−1s	t = 1−2s	t = 2−3s	t = 3−4s
MOD1	740–50	50–350	350	350–100
MOD2	808–250	250–550	550	550–350
MOD3	830–140	140–650	650	650–210
MOD4	850–231	231–750	750	750–321
MOD5	880–150	150–850	850	850–300
MOD6	900–300	300–880	880	880–400
MOD7	950–350	350–902	902	902–650
MOD8	980–450	450–950	950	950–750
MOD9	1000–550	550–1000	1000	1000–850
Array	7938–2470.5	2471–6882	6882	6882–3931

**Table 12:** Output power rating of 3 × 3 PV array under linearly changing irradiance profiles.

Irradiance (W/m^2^)	Power rating
7000	2868.74 W
1000	409.82 W
6000	2458.92 W
1850	758.17 W

In this situation, as the operating point is too far away from the reference operating point, so APO resulted in the overshoot due to large step sizes during transient response. However, due to a slow tracking response, it does not follow the reference trajectory as seen in Fig. 27a. The expected output power of the PV system is computed as 3253W. APO expressed overshoot at 3502.44W and then obtained 2721.69W output power at 0.43 s. The CMPC algorithm expressed fluctuations while achieving the GMPP as presented in Fig. 24b and achieved 3251W output power with oscillations of ± 2W in steady-state at 0.45s. The proposed algorithm illustrated smooth transient response by achieving 3253W output power at 0.22 s without oscillations during steady-state response.

Considering the output power at t = 0–1s as shown in Fig. 27a, the reference operating point is far away due to high irradiance on each module, therefore, APO causes overshoot at 3501W with slow convergence towards GMPP as presented in Fig. 27b. The CMPC tries to achieve the operating point but it causes many fluctuations. Finally, the proposed algorithm achieved GMPP very smoothly without considerable oscillations with respect to APO and CMPC.

In the case of linear rise in irradiance, the GMPP tracking of APO and CMPC is also somehow better as presented in Fig. 27c. Similarly, under linear variations in solar irradiance at t = 1s as illustrated in Fig. 27d, the proposed algorithm efficiently followed the reference operating point to track GMPP at 2820.5W. While linear decrease occurs in solar irradiance, APO-MPC and CMPC follow the reference trajectory, however, APO deviated from achieving GMPP as illustrated in Fig. 27e. Finally, we can observe in Fig. 28a that the proposed algorithm expressed more accurate and efficient behavior nearer to GMPP without considerable oscillations and any overshoot. The duty cycle behavior of all three algorithms is presented in Fig. 28b. A comparison of overall efficiency and error analysis in terms of deviation from the reference operating point are expressed in Fig. 28c,e respectively. Figure 28d illustrates the step response of all algorithms, from which, APO-MPC has minimum GMPP tracking time over other algorithms.

As observed in simulated results, the proposed methodology smoothly transits towards GMPP by comparing output power of each module instantly and evaluating the LMPPs. After computations it rejects all possible LMPPs except the one which has greater output power to avoid falling into any LMPP and follows the GMPP. Similarly, it initiates testing for both points, after which it returns to the first point because it is near the GMPP.

### Experimental testing

**Figure 29:**
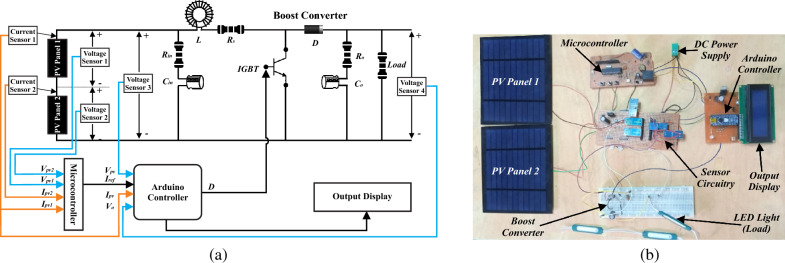
Overview of experimental setup (**a**) circuit diagram of the designed prototype (**b**) hardware based practical prototype.

To validate the effectiveness of APO-MPC practically, a hardware-based prototype comprises two PV panels, sensor circuitry to measure voltages and currents, a microcontroller to generate reference current, and a boost converter regulated by an Arduino controller. A light emitting diode (LED) is used as load and a multi segment liquid crystal display (LCD) is used as output display. The schematic circuit diagram and hardware implementation of experimental setup are illustrated in Fig. 29a,b respectively. The components description and protype working are expressed in detail as follows: The PV modules used to build this prototype are 2.5W OEM Solar Module WSL-C006 (monocrystalline solar cell) with nominal characteristics of V_mp_ = 5 V, V_oc_ = 6 V, I_mp_ = 500 mA, and I_sc_ = 660 mA at STC. For measuring output voltages and currents, F031-06 voltage sensors and ACS712 current sensors are utilized. For implementation of APO and MPC algorithms PIC16F877A and Arduino Nano ATmega328 microcontrollers are used. IRF630-N MOSFET is used as switching component, Sunfounder I2C LCD is embedded as display unit to watch output parameters and LED strip lights are used as load. The components used in boost converter are illustrated in Table 5.

**Figure 30:**
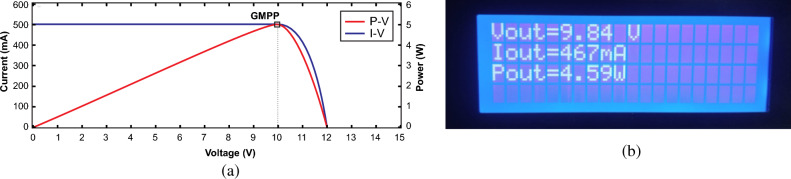
Output parameters under UIC (**a**) estimated GMPP values (**b**) experimental recorded values.

The experiment was conducted by exposing the hardware model in front of the sunlight at noon. First, both of the PV panels are exposed simultaneously to verify UIC scenario comprising estimated parameters at GMPP as G_1,2_ = 1000 W/m^2^, V_o_ = 10 V, I_o_ = 500 mA and P_o_ = 5 W and actual output parameters V_o_ = 9.84 V, I_o_ = 467 mA and P_o_ = 4.59 W were recorded as illustrated in Fig. 30a,b respectively. By measuring output current across each panel, the projected irradiance was estimated using Eq. (38) because the irradiance is directly linked to the current of the PV panel^50^. Due to similar irradiance on both panels, the algorithm expressed only single peak while tracking GMPP. The overall efficiency of the system under this scenario is recorded as 91.8%.

**Figure 31:**
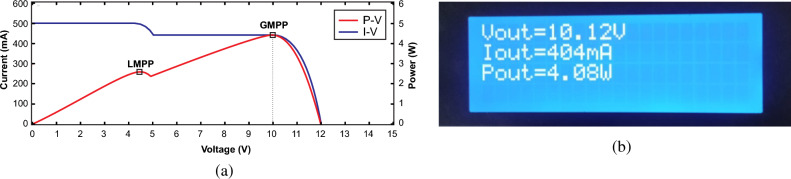
Output parameters under PSC1 (**a**) estimated GMPP values (**b**) experimental recorded values.

38 $$G=\frac{1000 \times {I}_{pv}}{{I}_{sc}}$$

For validation of PSC, a small portion of one panel was shaded using a card board comprising estimated parameters at GMPP as G_1_ = 1000 W/m^2^, G_2_ = 900 W/m^2^, V_o_ = 10 V, I_o_ = 445 mA and P_o_ = 4.45 W and actual output parameters V_o_ = 10.12 V, I_o_ = 404 mA and P_o_ = 4.08 W were recorded as illustrated in Fig. 31a,b respectively. In this case, the algorithm assessed the change in irradiance with change in output power on both panels, therefore, it expressed one LMPP and closely tracked the GMPP. The overall efficiency of the system under this scenario is recorded as 91.68%.

**Figure 32:**
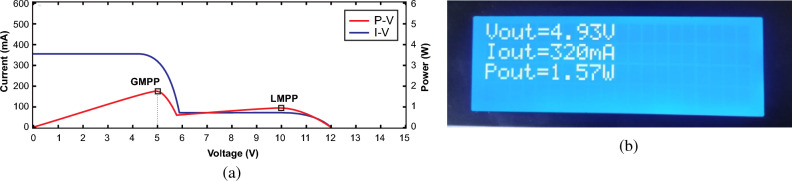
Output parameters under PSC2 (**a**) estimated GMPP values (**b**) experimental recorded values.

For further validation of PSC scenario, some portions of both panels were shaded using card boards comprising estimated parameters at GMPP as G_1_ = 700 W/m^2^, G_2_ = 200 W/m^2^, V_o_ = 5 V, I_o_ = 350 mA and P_o_ = 1.75 W and actual output parameters V_o_ = 4.93 V, I_o_ = 320 mA and P_o_ = 1.57 W were recorded as illustrated in Fig. 32a,b respectively. Similarly, the recorded values remain close to the GMPP and away from LMPP. The overall efficiency of the system under this scenario is recorded as 89.71%. According to the performance of hardware prototype under different scenarios, the proposed algorithm efficiently tracked the GMPP. However, the output parameters derived from the practical experiment display some variance from those computed through simulation, attributed to various factors such as sensor noise, communication speed, and the computational power.

### Comparative analysis

After assessing the overall performance validation, it is possible to determine the best technique for MPPT in PV systems as each algorithm has its advantages and disadvantages. The simulation results based average tracking efficiency of APO is 92.18%, CMPC has 98.05% and APO-MPC has 99.82%. Similarly, the proposed algorithm is compared with previously implemented P&O^27^ and MPC-MPPT^51^, which illustrates the tracking range under experimental results. P&O algorithm expressed the minimum performance efficiency of 87.36%, MPC presented 87–93.50% and APO-MPC has illustrated 89.71–91.80% under different scenarios. The variations between simulated and experimental results can be mitigated by using high quality sensors for measuring different parameters, enhancing the computational power of controller and communication speed. In this study, the comparative analysis based on performance and efficiency is presented in Table 13, and qualitative comparison based on their performance assigned with a grade is listed in Table 14.

**Table 13:** Comparative analysis of performance and efficiency.

Irradiance profile	Expected GMPP	APO-MPC		CMPC		APO	
		Tracked GMPP	Tracking efficiency	Tracked GMPP	Tracking efficiency	Tracked GMPP	Tracking efficiency
Case 1	1800.12 W	1794.53 W	99.68%	1759.65 W	97.75%	1664.75 W	92.47%
Case 2	1229.46 W	1228.11 W	99.89%	1211.59 W	98.54%	1167.11 W	94.92%
Case 3	2920.32 W	2917.24 W	99.89%	2887.46 W	98.87%	2604.01 W	89.16%
Average tracking efficiency (simulations)		99.82%		98.05%		92.18%	
Tracking range (experimental)		89.71–91.80%		87–93.50%^51^		87.36%^27^

**Table 14:** Qualitative comparison on the basis of different performance factors.

Algorithms	Exact MPPT	Regular adjusting	Tracking speed	Analog or digital	Complexity	Sensed variables	Climate	Grade
APO-MPC	Yes	No	Fast	Both	Medium	V, I	PSC/UIC	Best
CMPC	Yes/no	No	Fast/medium	Both	Medium	V, I	PSC/UIC	Good
APO	No	No	Medium/low	Single/both	Low	Varies	UIC	Average

## Conclusion

In this paper, an adapted method is introduced for optimizing the GMPP of a PV system by utilizing a hybrid APO-MPC control algorithm under various climatic profiles of PSCs. The APO algorithm computes reference current using variable step sizes. The performance of the proposed algorithm is evaluated and validated in comparison with APO and CMPC using MATLAB/Simulink and experimental validations to ensure its capability to successfully attain the GMPP even under dynamic weather scenarios. The modified boost converter is utilized to regulate voltage and current levels within the PV system. It is observed that the average tracking efficiency of the proposed algorithm is 99.82% by conducting various testing scenarios under dynamic weather profiles of PSCs. From the results, it can be concluded that the proposed APO-MPC method outperforms the APO and CMPC methods, particularly in terms of rapid tracking response, smooth steady-state response, absence of overshoots, and efficiently maintained GMPP tracking even under dynamic climates and PSCs.

## Data Availability

The data that support the findings of this study are available from the corresponding author on reasonable request.
