# Author Correction: An adapted model predictive control MPPT for validation of optimum GMPP tracking under partial shading conditions

**DOI:** 10.1038/s41598-024-62104-0

**Published:** 2024-05-15

**Authors:** Muhammad Abu Bakar Siddique, Dongya Zhao, Ateeq Ur Rehman, Khmaies Ouahada, Habib Hamam

**Affiliations:** 1 College of New Energy, China University of Petroleum (East China), Qingdao, 266580 China; 2 School of Computing, Gachon University, Seongnam, 13120 Republic of Korea; 3 Department of Electrical and Electronic Engineering Science, School of Electrical Engineering, University of Johannesburg, Johannesburg, 2006 South Africa; 4 Faculty of Engineering, Université de Moncton, Moncton, NB E1A3E9 Canada; 5 Hodmas University College, Taleh Area, Mogadishu, Somalia; 6 Bridges for Academic Excellence, Tunis, Centre‑Ville, Tunisia

Correction to: *Scientific Reports* 10.1038/s41598-024-59304-z, published online 24 April 2024

The original version of this Article contained an error in the spelling of the author Khmaies Ouahada which was incorrectly given as Khmeis Ouahada.

The original Article has been corrected.

